# Anticipated burden and mitigation of carbon-dioxide-induced nutritional deficiencies and related diseases: A simulation modeling study

**DOI:** 10.1371/journal.pmed.1002586

**Published:** 2018-07-03

**Authors:** Christopher Weyant, Margaret L. Brandeau, Marshall Burke, David B. Lobell, Eran Bendavid, Sanjay Basu

**Affiliations:** 1 Department of Management Science and Engineering, Stanford University, Stanford, California, United States of America; 2 Department of Earth System Science, Stanford University, Stanford, California, United States of America; 3 Center on Food Security and the Environment, Stanford University, Stanford, California, United States of America; 4 Center for Primary Care and Outcomes, Stanford University, Stanford, California, United States of America; 5 Center for Population Health Sciences, Stanford University, Stanford, California, United States of America; 6 Department of Medicine, Stanford University, Stanford, California, United States of America; 7 Department of Health Research and Policy, Stanford University, Stanford, California, United States of America; 8 Center for Primary Care, Harvard Medical School, Boston, Massachusetts, United States of America

## Abstract

**Background:**

Rising atmospheric carbon dioxide concentrations are anticipated to decrease the zinc and iron concentrations of crops. The associated disease burden and optimal mitigation strategies remain unknown. We sought to understand where and to what extent increasing carbon dioxide concentrations may increase the global burden of nutritional deficiencies through changes in crop nutrient concentrations, and the effects of potential mitigation strategies.

**Methods and findings:**

For each of 137 countries, we incorporated estimates of climate change, crop nutrient concentrations, dietary patterns, and disease risk into a microsimulation model of zinc and iron deficiency. These estimates were obtained from the Intergovernmental Panel on Climate Change, US Department of Agriculture, Statistics Division of the Food and Agriculture Organization of the United Nations, and Global Burden of Disease Project, respectively. In the absence of increasing carbon dioxide concentrations, we estimated that zinc and iron deficiencies would induce 1,072.9 million disability-adjusted life years (DALYs) globally over the period 2015 to 2050 (95% credible interval [CrI]: 971.1–1,167.7). In the presence of increasing carbon dioxide concentrations, we estimated that decreasing zinc and iron concentrations of crops would induce an additional 125.8 million DALYs globally over the same period (95% CrI: 113.6–138.9). This carbon-dioxide-induced disease burden is projected to disproportionately affect nations in the World Health Organization’s South-East Asia and African Regions (44.0 and 28.5 million DALYs, respectively), which already have high existing disease burdens from zinc and iron deficiencies (364.3 and 299.5 million DALYs, respectively), increasing global nutritional inequalities. A climate mitigation strategy such as the Paris Agreement (an international agreement to keep global temperatures within 2°C of pre-industrial levels) would be expected to avert 48.2% of this burden (95% CrI: 47.8%–48.5%), while traditional public health interventions including nutrient supplementation and disease control programs would be expected to avert 26.6% of the burden (95% CrI: 23.8%–29.6%). Of the traditional public health interventions, zinc supplementation would be expected to avert 5.5%, iron supplementation 15.7%, malaria mitigation 3.2%, pneumonia mitigation 1.6%, and diarrhea mitigation 0.5%. The primary limitations of the analysis include uncertainty regarding how food consumption patterns may change with climate, how disease mortality rates will change over time, and how crop zinc and iron concentrations will decline from those at present to those in 2050.

**Conclusions:**

Effects of increased carbon dioxide on crop nutrient concentrations are anticipated to exacerbate inequalities in zinc and iron deficiencies by 2050. Proposed Paris Agreement strategies are expected to be more effective than traditional public health measures to avert the increased inequality.

## Abstract 2

In a modeling study Christopher Weyant and colleagues predict reduced crop nutritional content and subsequent health disparities due to increased carbon dioxide levels caused by climate change.

## Author summary

**Why was this study done?:**

- Increasing atmospheric carbon dioxide concentrations are anticipated to decrease the zinc and iron concentrations of some crops.
- The disease burden induced by changes in zinc and iron concentrations in crops, and optimal mitigation strategies, are unknown.

**What did the researchers do and find?:**

- We developed a country-level microsimulation model of zinc and iron deficiency to project the health effects of zinc- and iron-deficiency-related diseases in terms of disability-adjusted life years accumulated over the period 2015 to 2050, worldwide.
- We estimated that carbon-dioxide-induced reductions in the zinc and iron concentrations of crops would be expected to induce approximately 125.8 million disability-adjusted life years globally over the period from 2015 to 2050, disproportionately affecting South-East Asian and sub-Saharan African countries.
- We also estimated that a climate mitigation strategy concordant with the Paris Agreement would avert a greater portion of this burden (approximately 48.2%) than traditional public health interventions (approximately 26.6%).

**What do these findings mean?:**

- The effects of increased carbon dioxide concentrations on crop nutrient concentrations may increase existing inequalities in nutritional deficiencies by 2050.
- Climate mitigation strategies, such as the Paris Agreement, may be more effective than traditional public health interventions in averting this increased inequality.

## Introduction

Increasing atmospheric carbon dioxide concentrations are anticipated to affect public health through increased unsafe weather events, extreme heat, air pollution, and allergen and disease vector exposure [1]. Increasing carbon dioxide concentrations are also anticipated to reduce the concentrations of zinc and iron in many agricultural crops, particularly C_3_ plants, which rely solely on C_3_ carbon fixation and include common rice and wheat varieties; C_3_ plants constitute 95% of terrestrial plants, account for over half of global caloric consumption, and serve as the primary source of dietary zinc and iron for populations at highest risk of nutritional deficiencies [2–5]. The empirical observation of lowered zinc and iron concentrations under elevated carbon dioxide is well established, although the mechanisms are not yet well understood and likely relate in part to changes in crop transpiration [2,5,6]. Zinc and iron deficiencies, in turn, increase the risk of infections, diarrhea, and anemia [7,8]. It remains unclear, however, where and to what degree increasing carbon dioxide concentrations may increase the global burden of nutritional deficiencies, and which strategies may best mitigate the increase. An active area of public health policy debate is whether direct climate change mitigation strategies, such as the Paris Agreement, will be sufficient or comparable to traditional public health measures to combat the complications of carbon-dioxide-induced zinc and iron deficiencies—particularly supplementation and disease control programs to combat the heightened risk of infections, diarrhea, and anemia [9].

The impact of increased carbon dioxide on nutritional deficiencies and their associated diseases depends on 5 related factors: the magnitude of increase in atmospheric carbon dioxide concentrations; the resultant changes in crop nutrient concentrations; current and future trends in consumption of crops and other dietary nutrient sources; the risks of deficiencies, given dietary consumption; and the risks of disease, given nutritional deficiencies.

**Fig 1:**
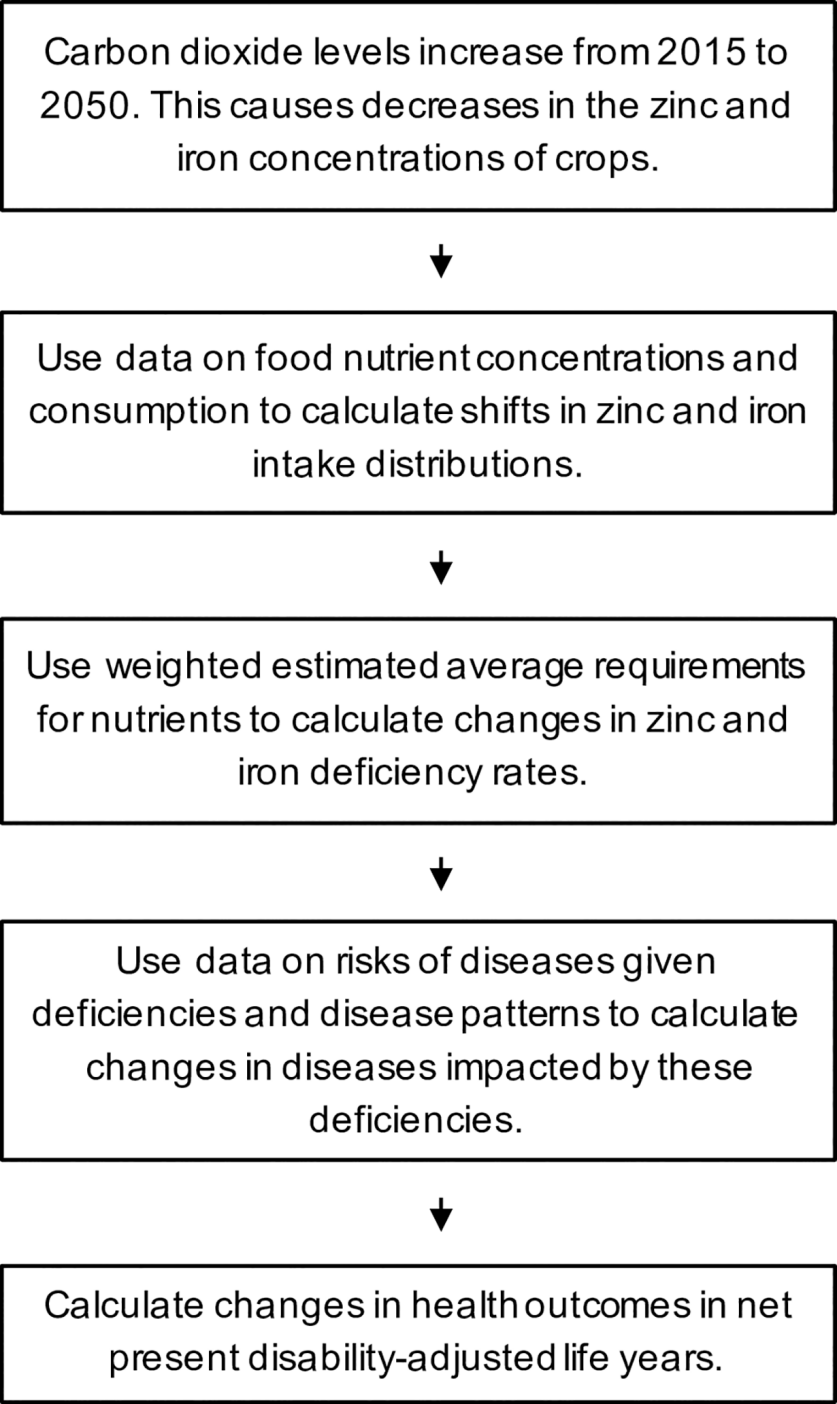
Model schematic. In the model, increased atmospheric carbon dioxide concentrations decrease the zinc and iron concentrations of crops. Data on food nutrient concentrations and consumption are used to calculate changes in zinc and iron intake distributions. These distributions and weighted estimated average requirements (WtdEARs) for nutrients are used as inputs to a microsimulation, which projects health outcomes due to carbon-dioxide-induced declines in the zinc and iron concentrations of crops.

We developed a model (Fig 1) to estimate where and to what degree increasing carbon dioxide concentrations would be expected to increase the global burden of zinc and iron deficiencies and their associated diseases over the period 2015 to 2050. We used this model to evaluate which strategies may best mitigate the burden of disease attributable to increased carbon dioxide in the atmosphere. Specifically, we created a microsimulation in which demographically representative populations in each country were or were not exposed to carbon-dioxide-induced changes in the zinc and iron concentrations of crops, and experienced related disease burdens quantified in disability-adjusted life years (DALYs).

## Methods

### Data sources

To estimate the impact of increasing carbon dioxide concentrations on nutritional deficiencies and associated disease rates, we used 5 main data sources. We focused our analysis on country-level estimates of the following: carbon dioxide concentration changes from the Intergovernmental Panel on Climate Change [10], crop zinc and iron concentrations from the US Department of Agriculture (USDA) [11], changes in crop zinc and iron concentrations due to increasing carbon dioxide concentrations from Myers et al. [5], dietary supply from the Statistics Division of the Food and Agriculture Organization of the United Nations (FAOSTAT) [12], and disease prevalence among different populations globally from the Global Burden of Disease Project (GBD) [13]. Example values for Nigeria are shown in Table 1, with links to primary data for other countries in S1 and S2 Texts.

**Table 1:** Model inputs, with example values from Nigeria.

Variable	Value	Units	Source
**Nutritional requirements**			
Zinc required per day (WtdEAR)	10.33	mg/capita/day	[14]
Iron required per day (WtdEAR)	18.91	mg/capita/day	[8,12]*
**Disease prevalence**			
Malaria prevalence	3.80 × 10^−2^	Fraction of population	[13,15]*
Pneumonia prevalence	7.42 × 10^−4^	Fraction of population	[13,15]*
Diarrhea prevalence	5.50 × 10^−2^	Fraction of population	[13,15]*
**Disability weights**			
Malaria weight	1.91 × 10^−1^	NA	[15]
Pneumonia weight	2.79 × 10^−1^	NA	[15]
Diarrhea weight	1.05 × 10^−1^	NA	[15]
Iron deficiency anemia weight	3.12 × 10^−2^	NA	[8,13]*
**Relative disease risks**			
RR malaria given zinc deficient	1.56	NA	[7]
RR pneumonia given zinc deficient	1.52	NA	[7]
RR diarrhea given zinc deficient	1.28	NA	[7]

*Calculated value.

NA, not applicable; RR, relative risk; WtdEAR, weighted estimated average requirement.

### Model structure

We incorporated the data into a microsimulation model (Fig 1) that simulates individuals within each country, reflecting the range of consumption of zinc and iron given the variations in dietary patterns in each country, and associated zinc and iron deficiency status and attendant risks for infections, diarrhea, and anemia. For each of 137 countries with data, we simulated 2 identical samples of 10,000 individuals demographically distributed to match country-specific age, sex, and secular birth/death trends. Each individual was probabilistically given values of zinc and iron consumption, morbidity risk, and mortality risk (Table 1; Fig 1). One population sample was subjected to carbon-dioxide-induced declines in the zinc and iron concentrations of crops while the other maintained current levels of zinc and iron concentrations. The primary model outcome was the difference between the 2 populations in DALYs per 1,000 persons. DALYs are the sum of years of life lost from disease and years of life lived with disability, weighted by the disability associated with each disease endpoint (Table 1).

In the simulation, we assumed that current dietary patterns (number of kilocalories/capita/day consumed of each food category) would remain the same despite increasing carbon dioxide concentrations, meaning that individuals would not adjust their total intake of food, or types of food eaten, based on reduction in nutrients in each crop. Average food consumption per capita in each country was determined from FAOSTAT data on approximately 100 mutually exclusive and collectively exhaustive food categories, such as potatoes, bovine meat, and beans [12]. The baseline zinc and iron contents per 100 grams and energy density of each food type were obtained from USDA data [11]. Country-specific zinc consumption distributions were constructed assuming normality and coefficients of variation (CVs) of 25% [16–18]. This method of constructing zinc consumption distributions from FAOSTAT and USDA data is similar to that used by Kumssa et al. [14]. Country-specific iron consumption distributions were also constructed assuming normality and CVs of 25% following prior literature [16]. An alternative assumption for iron consumption distributions, lognormality with CVs of 40% [19], was applied in sensitivity analysis (S4 Text). The annual crop-specific declines in zinc and iron concentrations due to increasing carbon dioxide were estimated from nutrient concentrations of crops grown under carbon dioxide concentrations expected by the year 2050 [5] with assumed linear declines in crop concentrations over the period from 2015 to 2050. The expected carbon dioxide concentrations by the year 2050 used in [5] are based on the Intergovernmental Panel on Climate Change’s assessment of carbon dioxide concentration projections [10], which are the same in all countries. Specifically, projected declines in the micronutrient concentrations of particular crops were extrapolated to broader crop categories, in keeping with previous studies (S1 Text) [9,20].

Given the annual level of zinc and iron consumed by each simulated individual, we determined whether an individual fell below a critical threshold level of zinc or iron intake that would be labeled “deficiency” and would predispose the individual to disease, using the estimated average requirement cut-point method [21–23], which compares a person’s average annual intake to the corresponding weighted estimated average requirement (WtdEAR). A WtdEAR is an average micronutrient requirement for a population and can be calculated by multiplying the requirement for each demographic group by the fraction of people in that demographic group and summing; the requirement for each demographic group is based on population-specific absorption factors [24].

The microsimulation model estimated the total morbidity and mortality burden associated with zinc and iron deficiencies per 1,000 people and for the overall population in each country. Zinc deficiency is associated with a heightened relative risk of morbidity and mortality from malaria, pneumonia, and diarrhea among children under 5 years, with no heightened disease risks among older persons [7]; the disability weights attributable to each of these conditions were obtained from prior assessments (Table 1) [8,13,15]. Prevalence rates, mortality rates, and trends over time for malaria, pneumonia, and diarrhea in each country were obtained from the GBD [13]. Mortality trends assumed continuation of the annual percent change in the per capita mortality for each disease as calculated by the GBD. The GBD calculated these values based on historical data from 1990 to 2013. Similarly, disease prevalence trends assumed continuation of the annual percent change in the prevalence rate for each disease from 1990 to 2013 as obtained from GBD data; in sensitivity analysis, disease prevalence rates were held constant (S8 Table). Iron deficiency was simulated as producing associated anemia, with disability weights, mortality rates, and mortality trends over time also from GBD data [8,13]. We note that iron deficiency and iron deficiency anemia (IDA) are not equivalent, but because IDA occurs when iron deficiency is sufficiently severe to reduce red blood cell production [25,26], the estimated average requirement cut-point method is a viable strategy for estimating the relative increase in IDA [27].

### Model validation

Validity of model estimates was assessed by comparing model estimates to values reported in the literature [8,13,14] (S1–S3 Tables). Model estimates subjected to validation included current zinc and iron dietary supplies and percent deficiencies, current yearly disease burdens, and projected disease burdens from 2015 to 2050 (calculated from current yearly burdens and assuming continuation of secular trends in the scenario without climate change).

### Comparative effectiveness

We considered alternative strategies to mitigate the disease burden from increased zinc and iron deficiencies attributable to increasing carbon dioxide concentrations. Specifically, we compared 5 traditional public health strategies to strategies consistent with the Paris Agreement to maintain global temperatures within 2°C of pre-industrial levels, adjusting each strategy for current anticipated feasibility and population reach. The 5 public health strategies were (i) zinc supplementation [28], in which zinc is administered daily to cover 80% of children under 5 years of age with random selection; (ii) iron supplementation [29], in which iron is administered weekly to cover 80% of females over 5 years of age; (iii) malaria mitigation [30], in which a previously described portfolio of interventions (e.g., long-lasting insecticide-treated nets, indoor residual spraying, and mass screening and treatment) is adopted at an 80% coverage level; (iv) pneumonia mitigation [31], in which a previously described portfolio of interventions (e.g., *Haemophilus influenzae* type b vaccine, pneumococcal vaccine, antibiotics for pneumonia, promotion of breastfeeding, vitamin A supplementation, and environmental improvements) is adopted at an 80% coverage level (except for the *H*. *influenzae* and pneumococcal vaccines, which are adopted at 90% coverage); and (v) diarrhea mitigation [31], in which a previously described portfolio of interventions (e.g., oral rehydration solution, rotavirus vaccine, antibiotics for dysentery, promotion of breastfeeding, vitamin A supplementation, and environmental improvements) is adopted at an 80% coverage level (except for the rotavirus vaccine, which is adopted at 90% coverage). Each strategy is detailed in S5 Text.

### Sensitivity analyses

We performed 1-way sensitivity analysis, in which parameters were varied one at a time ± 10%, across all model inputs (S4 Text; S2–S8 Figs). In particular, we varied initial zinc and iron intake distributions across ranges to account for potential changes in dietary patterns and uncertainties in the quality of current measurements, initial disease prevalence rates to account for potential underreporting, and disease mortality rates conditional on deficiency to account for potential changes in healthcare availability. We also performed probabilistic sensitivity analysis by running the model 10,000 times per country while Monte Carlo sampling from probability distributions around each input parameter to compute uncertainty intervals for each outcome (S4 Text; S5–S8 Tables). Probability distributions for input parameters were determined based on previously reported probability distributions, uncertainty estimates, and natural bounds. The model was programmed in R version 3.2.2 [32], with input data and statistical code for replication and extension of our analysis published at https://purl.stanford.edu/tx325yy8269 concurrent with publication.

## Results

### Model validation

Our model-based estimates of nutrient supply, nutrient deficiency, and associated disease had a high degree of correspondence to current estimates from other sources (S1–S3 Tables). Specifically, our model-based estimates of global population-weighted zinc supply and deficiency were within 0.8% and 3.3% relative error from values in the literature, respectively [14]. Our estimate of global population-weighted iron deficiency was within 0.6% of values in the literature [8]. Our estimates of the 2015 DALY burdens due to malaria, pneumonia, diarrhea, and IDA were within 2.4%, 0.3%, 1.4%, and 0.2%, respectively, of values in the literature [13]. Estimated 2015 to 2050 DALY burdens due to malaria, pneumonia, diarrhea, and IDA globally were within 0.7%, 1.3%, 2.0%, and 2.7%, respectively, of values in the literature [13]. All other model-based results showed high concordance with prior estimates of current nutrient deficiency and disease (S1–S3 Tables).

### Changes in zinc and iron deficiencies

In the absence of increasing carbon dioxide concentrations, zinc and iron deficiencies would be expected to induce 1,072.9 million DALYs globally over the period 2015 to 2050 according to our model. This global burden would represent approximately 2.0% of the expected DALYs due to all diseases of any kind over this period, which would make zinc and iron deficiencies the 13th leading risk factor for DALYs if the relative burdens attributable to other risk factors did not change [33].

**Fig 2:**
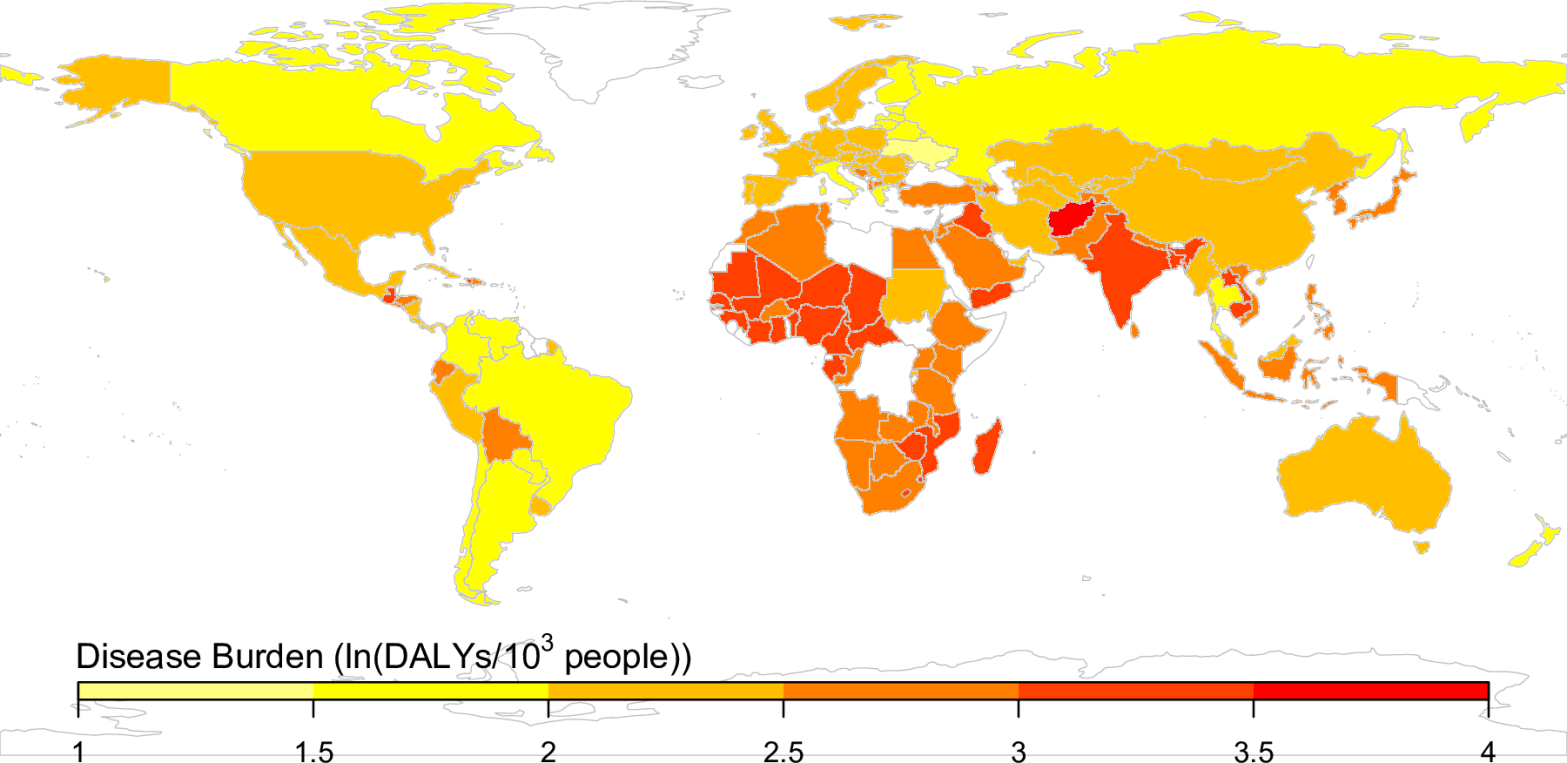
Per capita burden of disease attributed to carbon-dioxide-induced declines in the zinc and iron concentrations of crops, from 2015 to 2050. The microsimulation was run 10,000 times for each country, and the natural logarithm of burden in disability-adjusted life years (DALYs)/10^3^ people is shown. These values reflect the additional zinc and iron deficiency burden of disease due to increasing carbon dioxide, and background disease burdens are subtracted. Countries in white were those for which data were not available from the cited sources.

In the presence of increasing carbon dioxide concentrations, decreased zinc and iron concentrations of crops would be expected to lead to an additional 125.8 million DALYs globally over the period 2015 to 2050, with a disproportionate burden in the World Health Organization’s South-East Asia and African Regions (Fig 2). Countries in the study sample would be expected to experience increases in carbon dioxide concentration from approximately 400 ppm to 550 ppm (a 37.5% increase) from 2015 to 2050 and resultant declines in zinc and iron concentrations of C_3_ crops of approximately 5%–10% [5,9,10,34]. Given current consumption patterns, per our model, the nutrient concentration changes would induce an additional 8.2 percentage point increase in zinc deficiency (from the current baseline rate of 32.0% of the population deficient) and 6.1 percentage point increase in iron deficiency (from the baseline of 21.8% deficient) in the South-East Asia Region—the most highly affected region for zinc/iron deficiency—by 2050. The least-affected region, by contrast, would be the European Region, which would be anticipated to experience an additional 1.9 percentage point increase in zinc deficiency (from a baseline of 3.7% deficient) and 3.4 percentage point increase in iron deficiency (from a baseline of 11.1% deficient). The difference in the fraction of people placed at risk of zinc deficiency was not primarily due to a greater dependence on C_3_ crops in the South-East Asia Region for dietary zinc, but because a higher portion of the current population is only marginally above the threshold for zinc deficiency.

**Fig 3:**
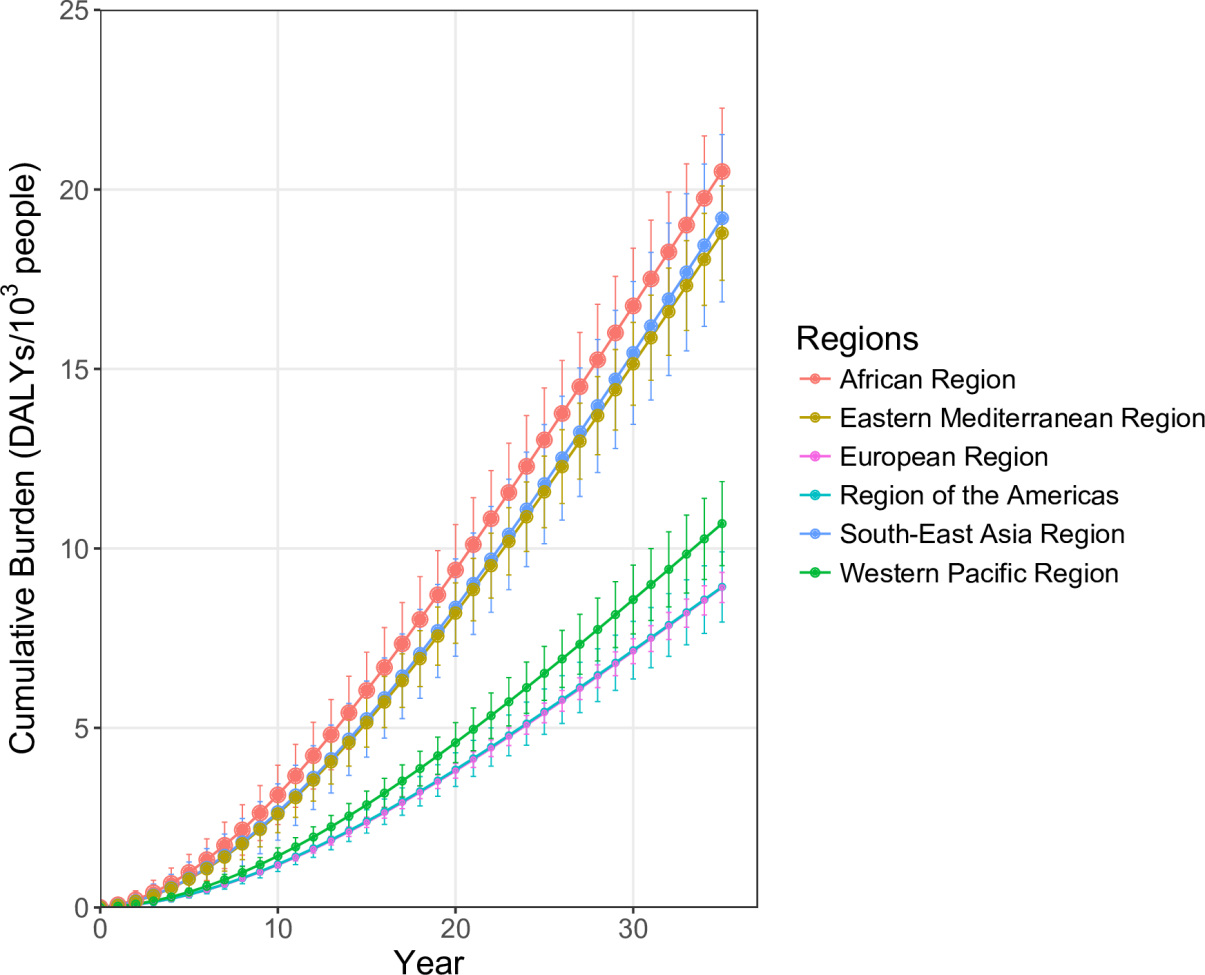
Cumulative per capita burden of disease attributed to carbon-dioxide-induced declines in the zinc and iron concentrations of crops, 2015 to 2050. The points show mean cumulative disability-adjusted life years (DALYs) per 1,000 population, with error bars reflecting 1 standard deviation above and below the mean from 10,000 probabilistic simulations. The size of the points for each population is scaled to show initial DALY burden per 1,000 population in 2015 due to zinc and iron deficiencies. Regions are as defined by WHO.

Model results suggested that carbon-dioxide-induced reductions in zinc and iron concentrations among crops would increase between-country and between-region inequalities in DALY burden per person (Fig 3). The regions with highest initial per capita burdens due to carbon-dioxide-induced zinc and iron reductions would also be expected to be the most affected over the 35-year period, with increased disparities over time. For example, the WHO African Region would be expected to experience approximately twice the per capita burden of other regions, such as the European Region, over the study period. Moreover, the regions with the highest per capita burdens due to carbon-dioxide-induced reductions (WHO African and South-East Asia Regions) are those with the highest current estimated burdens due to zinc and iron deficiencies (e.g., the current per capita burden of zinc and iron deficiencies in the African Region is approximately 4 times that of the European Region).

### Mitigation strategies

**Fig 4:**
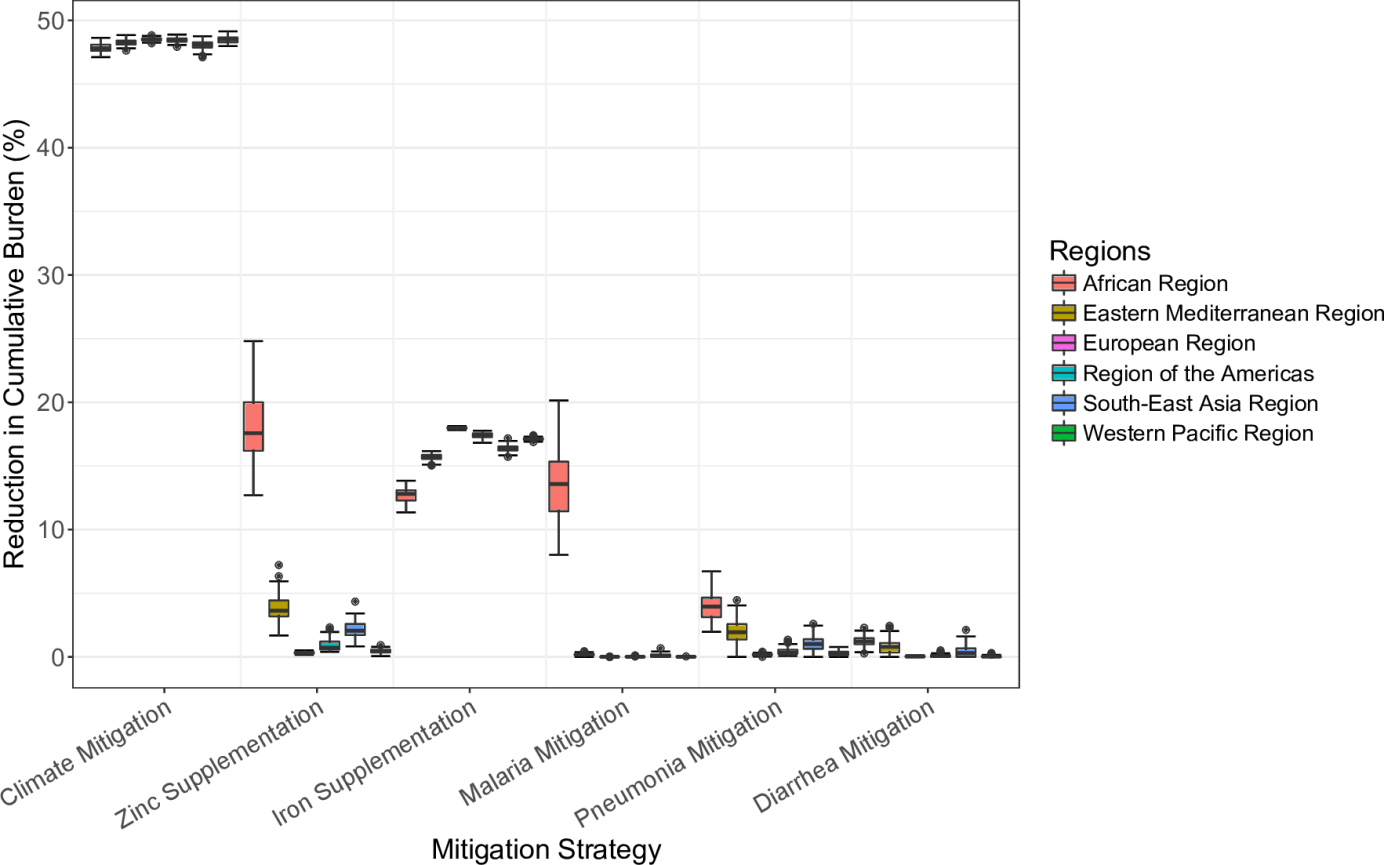
Effect of mitigation strategies, 2015 to 2050. Six mitigation strategies were considered for each of 137 countries. They included climate mitigation per the Paris Agreement to keep global temperatures below 2°C of pre-industrial levels; daily zinc supplementation for 80% of children under 5 years of age; weekly iron supplementation for 80% of women; and malaria, pneumonia, and diarrhea mitigation programs previously described in the literature adopted at 80% coverage levels (S5 Text). The microsimulation of 1 million people was run 100 times per country per strategy. The boxplots show median, interquartile, and outlier reductions in cumulative carbon-dioxide-induced disability-adjusted life year burdens from 2015 to 2050 for countries in each region by strategy. Of note, while there is considerable variation in carbon-dioxide-induced disease burden between iterations (Fig 3), there is relatively little variation in reduction in cumulative burden. This lack of variation is caused by the fact that all the mitigation strategies effectively reduce deficiencies or diseases on a percent basis (see Methods). Regions are as defined by WHO.

Compared with the 5 modeled traditional public health interventions, a climate mitigation strategy such as the Paris Agreement would be expected to avert the greatest portion of the cumulative projected burden of carbon-dioxide-attributable zinc and iron deficiencies between 2015 and 2050 (Fig 4). Even with 80% coverage with zinc and iron supplementation and with intensive disease control programs for malaria, pneumonia, and diarrhea, the sum total of the public health measures would be anticipated to reduce only 26.6% of the global carbon-dioxide-attributable zinc and iron deficiency burden of disease (95% credible interval [CrI]: 23.8%–29.6%). The countries that benefited most from the public health programs were those with high current zinc and iron deficiency DALY burdens (e.g., countries in the African Region), as these countries are expected to experience the greatest increases in deficiencies and highest disease rates over the study period.

By contrast, interventions consistent with the Paris Agreement to keep global temperatures within 2°C of pre-industrial levels, which was previously anticipated to feasibly prevent approximately 47% of the increase in carbon dioxide concentrations by 2050 [35,36], would be expected to avert 48.2% of global carbon-dioxide-attributable zinc and iron deficiency burden of disease (95% CrI: 47.8%–48.5%). Since the 2015 carbon dioxide concentration was approximately 400 ppm and the 2050 carbon dioxide concentration is projected to be approximately 550 ppm [5,9,10,34], adhering to the Paris Agreement would be expected to result in a 2050 carbon dioxide concentration of approximately 480 ppm.

The greater effectiveness of the climate mitigation strategy relative to the public health measures was driven by the fact that it benefited the entire population, whereas the public health measures were targeted to subsets of the population with the highest per capita DALY burden, missing substantial populations with small per capita, but large aggregate, DALY burdens. Furthermore, the Paris Agreement reversed carbon-dioxide-induced deficiencies and related diseases, unlike the public health measures, which each addressed only a single form of deficiency or disease manifestation. The credible intervals for the percent reductions in disease burdens with the mitigation strategies were relatively small as all the mitigation strategies effectively reduce deficiencies or related diseases on a percent basis.

### Sensitivity analyses

The relative benefits of the climate mitigation strategy versus the traditional public health strategies were unaltered by using a wide range of alternative literature-based input parameters (S2–S8 and S10 Figs; S7 and S8 Tables). In particular, the relative superiority of the climate mitigation strategy did not qualitatively change with alterations to baseline zinc and iron intakes, disease prevalence, or mortality rates. Model projections were generally most sensitive to changes in inputs related to iron (e.g., iron consumption, WtdEARs for iron, and IDA disability weights; S2–S8 Figs), but no scenario altered the comparative effectiveness of climate mitigation relative to traditional public health measures.

## Discussion

Carbon-dioxide-induced reductions in zinc and iron concentrations among crops are anticipated, by our model, to lead to an additional 125.8 million DALYs globally over the period 2015 to 2050. A disproportionate burden of these DALYs is anticipated to affect countries with high existing burdens from zinc and iron deficiencies, thereby increasing existing global inequalities in nutritional deficiency by disproportionately affecting South-East Asia and Africa.

Our study suggests that climate mitigation strategies such as the Paris Agreement would be expected to avert a greater portion (approximately 48%) of the projected increase in zinc and iron deficiencies, about 1.8 times greater than the mitigation from traditional public health interventions including nutrient supplementation and disease control programs to counteract the heightened risk of diseases associated with zinc and iron deficiencies. A 48.2% reduction in DALY burden due to reduced zinc and iron concentrations of crops with the climate mitigation strategy is reasonable, as the climate mitigation strategy reduces the increase in 2050 carbon dioxide concentrations by approximately 47%, and a linear relationship between carbon dioxide concentrations and plant micronutrient concentrations was assumed. However, this linear relationship need not be the case and is not an obvious result due to potential nonlinearities created by relationships between micronutrient supplies, deficiencies, diseases, and resulting DALY burdens.

Our study extends prior work projecting changes in the zinc and iron concentrations of crops due to increasing carbon dioxide concentrations, changes in the prevalence of zinc deficiencies, and countries at risk from the changing iron concentrations of crops (by computing changes in iron supplies and cross-referencing them to estimates of the current prevalence of anemia) [2–5,9,20]. We extended this body of work by modeling diseases stemming from zinc and iron deficiencies using a microsimulation model to project disease burdens attributable to the nutrient concentration effects of increased atmospheric carbon dioxide and to evaluate potential mitigation strategies. Critically, this approach enabled us to understand this important health impact of climate change in the context of existing disease burdens and health inequalities between countries and regions.

Among the primary limitations of our analysis is our inability to anticipate what changes in dietary patterns might occur simultaneously with increasing carbon dioxide concentrations. We varied consumption parameters over wide ranges in sensitivity analysis and found that our findings of an overall increased inequality in disease burden between countries and the impact of climate mitigation relative to public health measures were consistent across a broad range of scenarios. This is an important area for further research. Another limitation is that we assumed that secular exponential trends in disease mortality rates would continue, reflecting economic development and health services improvement. Should recent trends not continue in positive directions, our projections may be viewed as optimistic, rendering our results conservative forecasts of the complications from zinc and iron deficiencies. A third limitation is that we assumed that crop zinc and iron concentrations decline linearly with increasing carbon dioxide concentrations. If the declines are convex, our disease burden projections are likely underestimates, while if they are concave, our disease burden projections are likely overestimates. Finally, because we used WtdEARs to project deficiencies, we felt that disaggregating results by gender and age group would imply a level of precision we cannot reliably provide at this point.

Future studies may directly experiment with interventions to induce higher zinc and iron concentrations in plants that can be grown in the most heavily affected countries. Further studies should also evaluate whether dietary changes may be directed to reduce the burden of disease associated with nutritional deficiency attributable to increased carbon dioxide in the atmosphere. Finally, subsequent studies might seek to evaluate the cost and cost-effectiveness of different mitigation strategies.

While such studies are underway, our findings indicate that rising atmospheric carbon dioxide concentrations and their impacts on nutrient concentrations in crops are likely to increase health inequalities from nutritional deficiencies by disproportionately impacting countries with the highest existing health burdens attributable to nutritional deficiencies. This projected increase in health inequalities is driven by differences in existing vulnerability of populations marginally above the threshold for deficiency and associated disease risk. Mitigation strategies concordant with the Paris Agreement, however, would be anticipated to substantially reduce the carbon-dioxide-induced rise in zinc and iron deficiency burdens, as compared to traditional public health measures, which may have a lesser effect even with optimistic degrees of population coverage and efficacy.

## Supporting information

S1 Fig Modeled countries. Modeled countries are shown in color corresponding to their WHO region.
(TIF)

S2 Fig Results of 1-way sensitivity analysis for global DALY burden due to carbon-dioxide-induced declines in the zinc and iron concentrations of crops from 2015 to 2050. One-way sensitivity analysis was performed across all model inputs. For each country, inputs were varied by plus and minus 10%, and the microsimulation was run 1,000 times. The 6 most influential inputs globally are shown.
(TIF)

S3 Fig Results of 1-way sensitivity analysis for DALY burden in the African Region due to carbon-dioxide-induced declines in the zinc and iron concentrations of crops from 2015 to 2050. One-way sensitivity analysis was performed across all model inputs. For each country, inputs were varied by plus and minus 10%, and the microsimulation was run 1,000 times. The 6 most influential inputs are shown for the African Region.
(TIF)

S4 Fig Results of 1-way sensitivity analysis for DALY burden in the Region of the Americas due to carbon-dioxide-induced declines in the zinc and iron concentrations of crops from 2015 to 2050. One-way sensitivity analysis was performed across all model inputs. For each country, inputs were varied by plus and minus 10%, and the microsimulation was run 1,000 times. The 6 most influential inputs are shown for the Region of the Americas.
(TIF)

S5 Fig Results of 1-way sensitivity analysis for DALY burden in the South-East Asia Region due to carbon-dioxide-induced declines in the zinc and iron concentrations of crops from 2015 to 2050. One-way sensitivity analysis was performed across all model inputs. For each country, inputs were varied by plus and minus 10%, and the microsimulation was run 1,000 times. The 6 most influential inputs are shown for the South-East Asia Region.
(TIF)

S6 Fig Results of 1-way sensitivity analysis for DALY burden in the European Region due to carbon-dioxide-induced declines in the zinc and iron concentrations of crops from 2015 to 2050. One-way sensitivity analysis was performed across all model inputs. For each country, inputs were varied by plus and minus 10%, and the microsimulation was run 1,000 times. The 6 most influential inputs are shown for the European Region.
(TIF)

S7 Fig Results of 1-way sensitivity analysis for DALY burden in the Eastern Mediterranean Region due to carbon-dioxide-induced declines in the zinc and iron concentrations of crops from 2015 to 2050. One-way sensitivity analysis was performed across all model inputs. For each country, inputs were varied by plus and minus 10%, and the microsimulation was run 1,000 times. The 6 most influential inputs are shown for the Eastern Mediterranean Region.
(TIF)

S8 Fig Results of 1-way sensitivity analysis for DALY burden in the Western Pacific Region due to carbon-dioxide-induced declines in the zinc and iron concentrations of crops from 2015 to 2050. One-way sensitivity analysis was performed across all model inputs. For each country, inputs were varied by plus and minus 10%, and the microsimulation was run 1,000 times. The 6 most influential inputs are shown for the Western Pacific Region.
(TIF)

S9 Fig Cumulative per capita burden of disease attributed to carbon-dioxide-induced declines in the zinc and iron concentrations of crops by decadal period. The microsimulation was run 10,000 times for each country. The boxplots show median, interquartile, and outlier cumulative per capita carbon-dioxide-induced DALY burdens by decadal period for the countries in each region. These values reflect the additional zinc and iron deficiency burden of disease due to increasing carbon dioxide, and background disease burdens are subtracted.
(TIF)

S10 Fig Effects of mitigation strategies: percentage reduction in cumulative DALY burdens. The microsimulation of 1 million people was run 100 times per country per mitigation strategy. The boxplots show median, interquartile, and outlier reductions in cumulative DALY burdens from 2015 to 2050 for countries by region by strategy.
(TIF)

S1 Table Validation of zinc and iron dietary supplies and percent deficiencies in 2015. Results are shown as percent changes from literature values to model results. Iron supply could not be validated at the regional level as literature values were not available.
(DOCX)

S2 Table Validation of DALYs from malaria, pneumonia, diarrhea, and IDA in 2015. Results are shown as percent changes from literature values to model results.
(DOCX)

S3 Table Validation of DALYs from malaria, pneumonia, diarrhea, and IDA from 2015 to 2050. Results are shown as percent changes from literature values to model results.
(DOCX)

S4 Table Select model results by country. The microsimulation of 10,000 people was run 10,000 times for each country. Projected raw and per capita DALY burdens due to carbon-dioxide-induced declines in the zinc and iron concentrations of crops from 2015 to 2050 are shown. Micronutrient supplies and percent deficiencies in 2015 are also shown.
(DOCX)

S5 Table Distributions used in probabilistic sensitivity analysis. (DOCX)

S6 Table Results of probabilistic sensitivity analysis for DALY burden due to carbon-dioxide-induced declines in the zinc and iron concentrations of crops from 2015 to 2050. (DOCX)

S7 Table Sensitivity analysis with alternative iron consumption distributions. Iron consumption distributions were assumed to be lognormal with 40% CVs. The model was run 10,000 times with 10,000 people while sampling from distributions reflecting uncertainty in inputs.
(DOCX)

S8 Table Sensitivity analysis with constant disease prevalence rates. Malaria, pneumonia, and diarrhea prevalence rates in 2015 were assumed to continue over the model period. The model was run 10,000 times with 10,000 people while sampling from distributions reflecting uncertainty in inputs.
(DOCX)

S9 Table Select mitigation results by country. The microsimulation of 1 million people was run 100 times per country per mitigation strategy. Six mitigation strategies were considered. They included climate mitigation per the Paris Agreement to keep global temperatures below 2°C of pre-industrial levels; daily zinc supplementation for 80% of children under 5 years of age; weekly iron supplementation for 80% of women; and malaria, pneumonia, and diarrhea mitigation programs previously described in the literature adopted at 80% coverage rates. The mean percent reductions in cumulative DALY burdens due to carbon-dioxide-induced declines in zinc and iron concentrations of crops from 2015 to 2050 are shown.
(DOCX)

S1 Text Model inputs. (DOCX)

S2 Text Model structure. (DOCX)

S3 Text Model validation. (DOCX)

S4 Text Sensitivity analysis. (DOCX)

S5 Text Mitigation strategies. (DOCX)

## Abbreviations

CrI: credible interval
CV: coefficient of variation
DALY: disability-adjusted life year
GBD: Global Burden of Disease Project
IDA: iron deficiency anemia
USDA: US Department of Agriculture
WtdEAR: weighted estimated average requirement
