# Supplementary figures and images for: Anticipated burden and mitigation of carbon-dioxide-induced nutritional deficiencies and related diseases: A simulation modeling study
Source: PLoS Med. 2018 Jul 3;15(7):e1002586. doi: 10.1371/journal.pmed.1002586 (PMC6029750; doi:10.1371/journal.pmed.1002586)

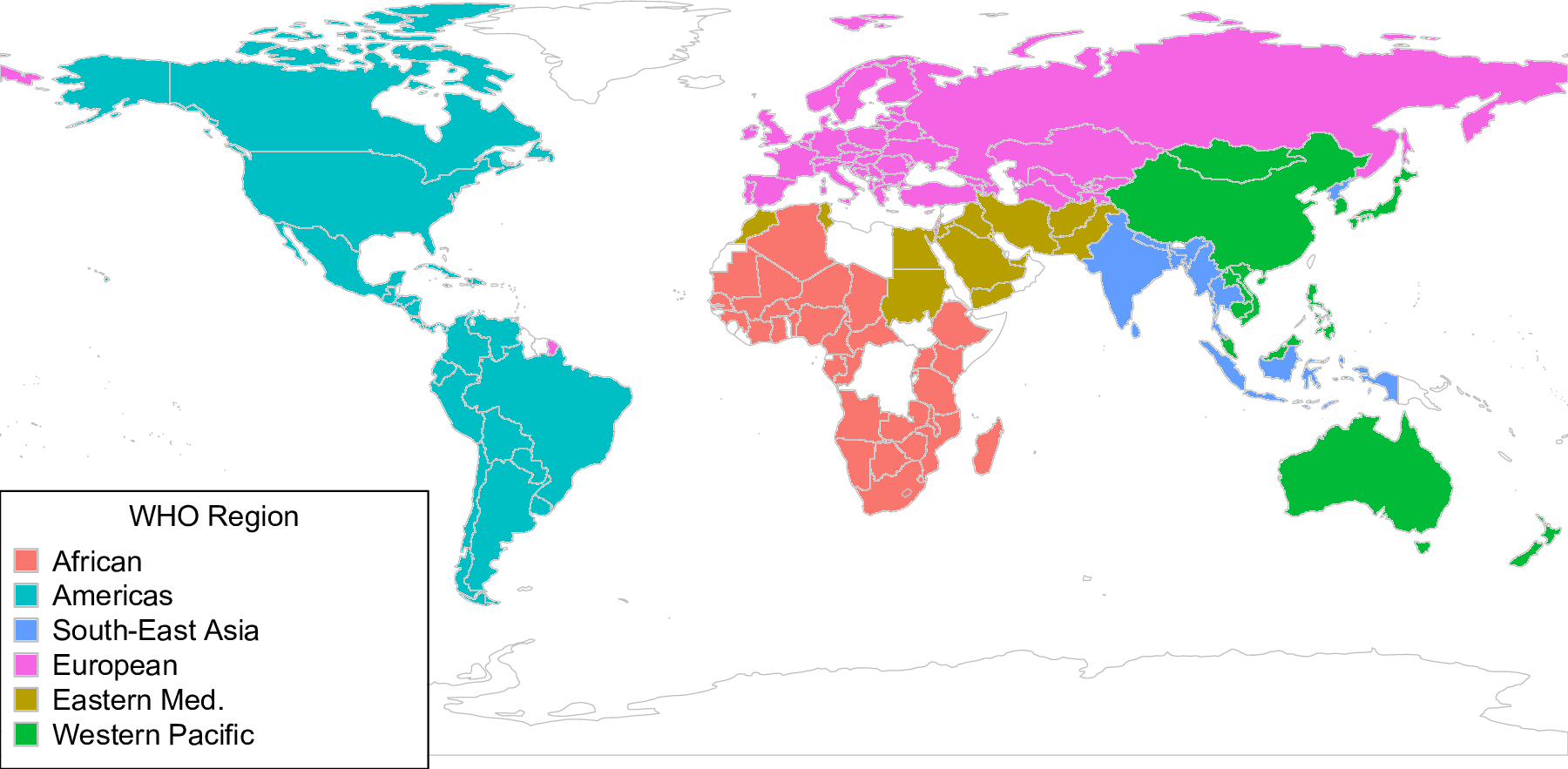

Supplement: S1 Fig — Modeled countries are shown in color corresponding to their WHO region. (TIF) [file pmed.1002586.s001.tif]

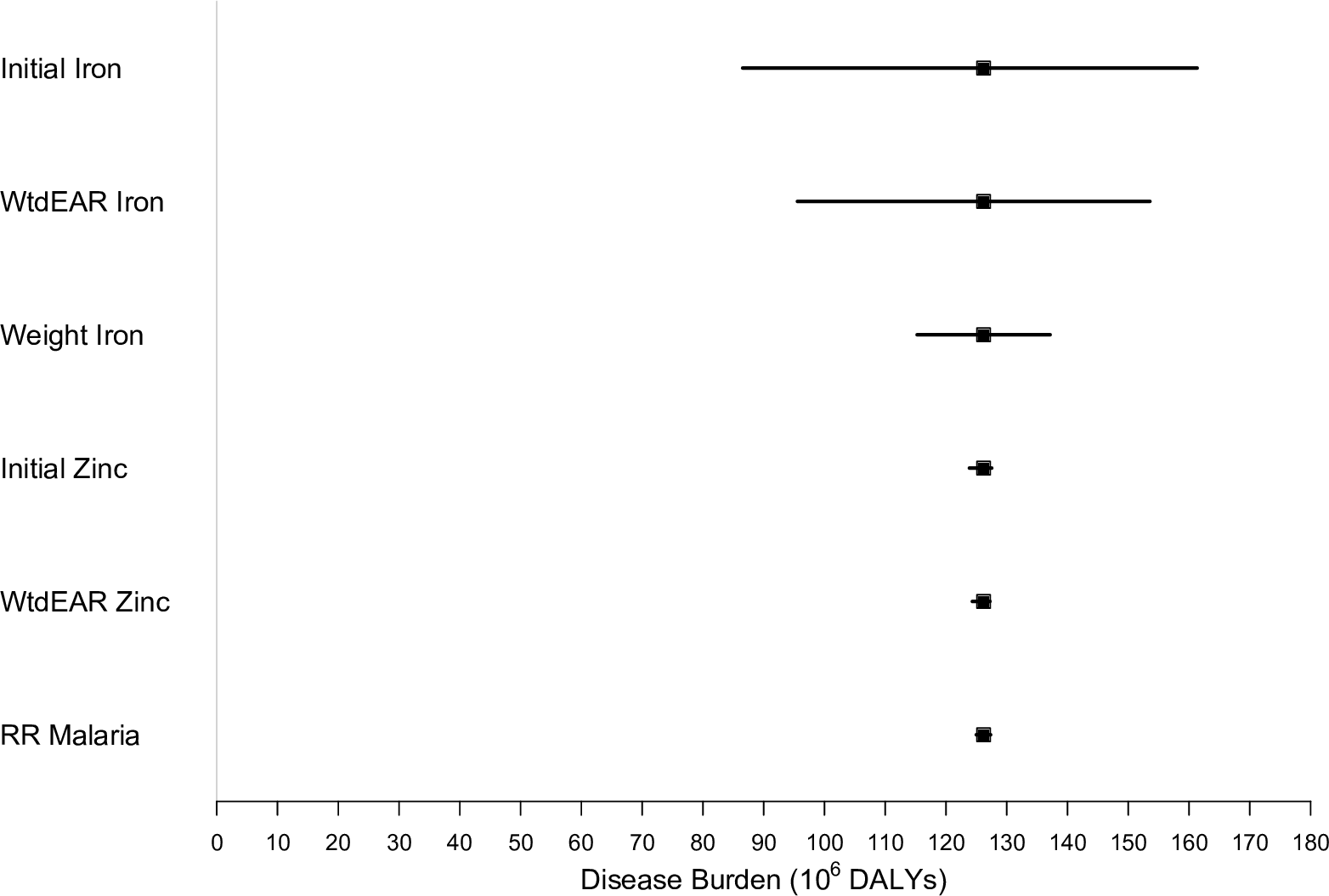

Supplement: S2 Fig — One-way sensitivity analysis was performed across all model inputs. For each country, inputs were varied by plus and minus 10%, and the microsimulation was run 1,000 times. The 6 most influential inputs globally are shown. (TIF) [file pmed.1002586.s002.tif]

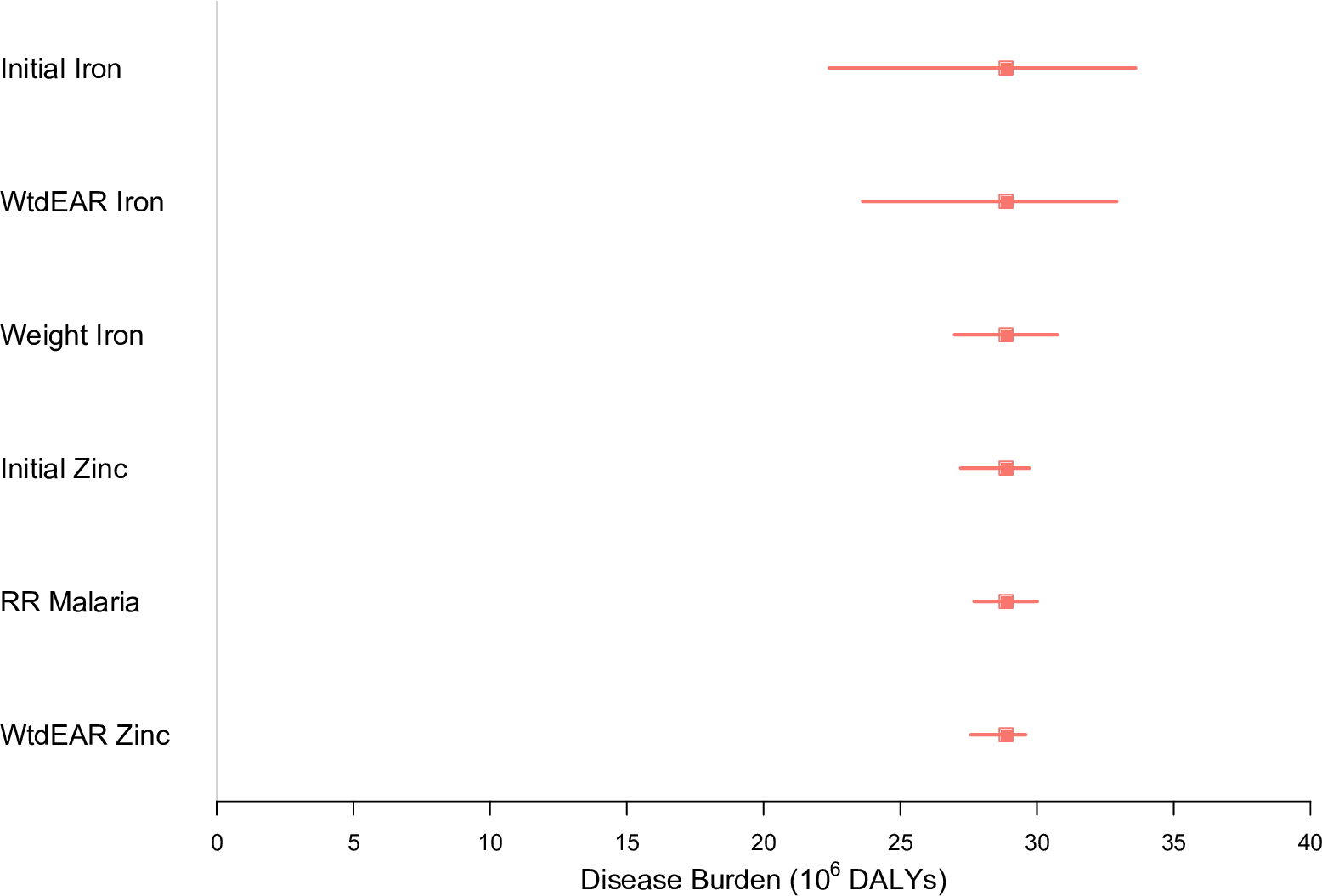

Supplement: S3 Fig — One-way sensitivity analysis was performed across all model inputs. For each country, inputs were varied by plus and minus 10%, and the microsimulation was run 1,000 times. The 6 most influential inputs are shown for the African Region. (TIF) [file pmed.1002586.s003.tif]

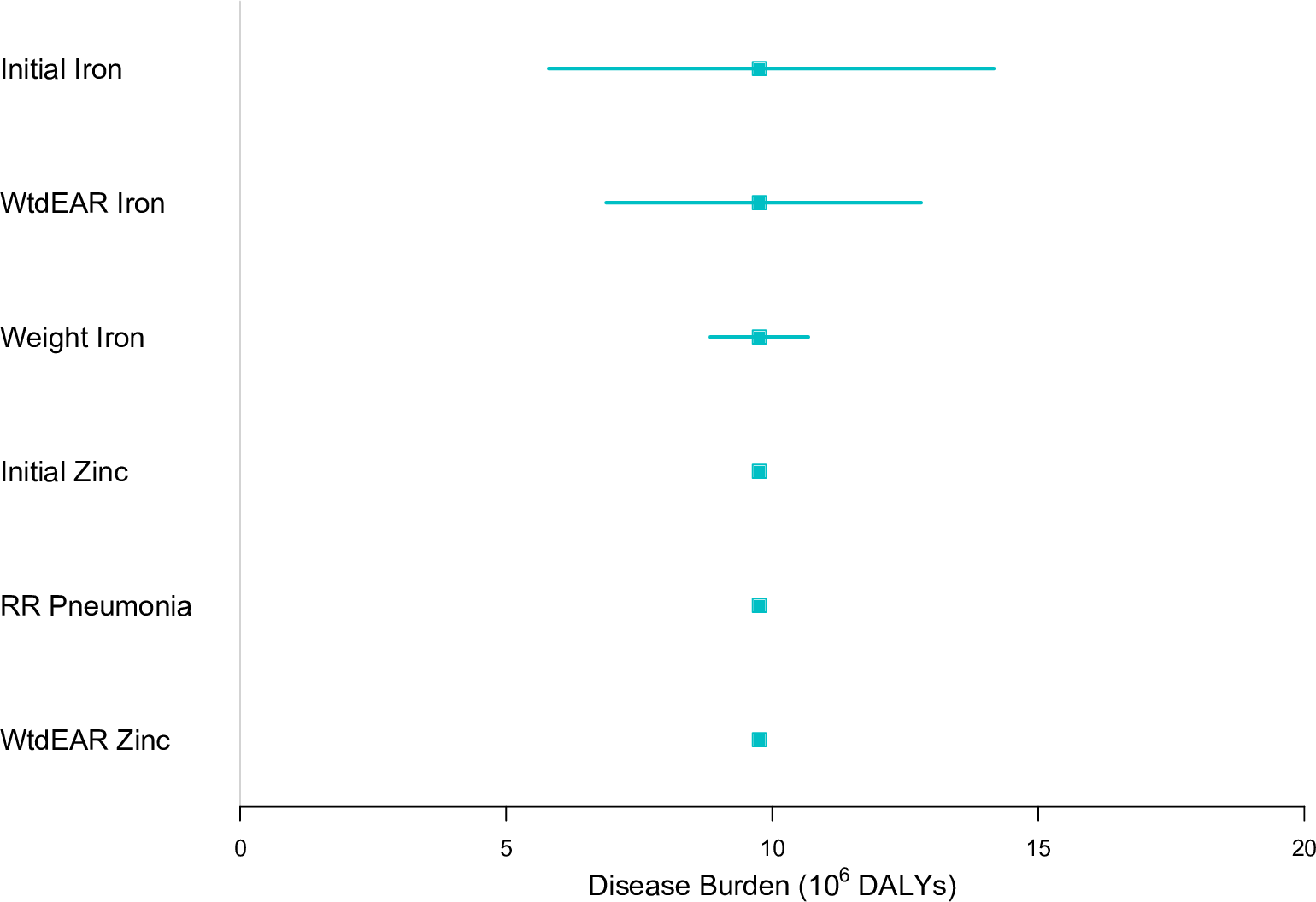

Supplement: S4 Fig — One-way sensitivity analysis was performed across all model inputs. For each country, inputs were varied by plus and minus 10%, and the microsimulation was run 1,000 times. The 6 most influential inputs are shown for the Region of the Americas. (TIF) [file pmed.1002586.s004.tif]

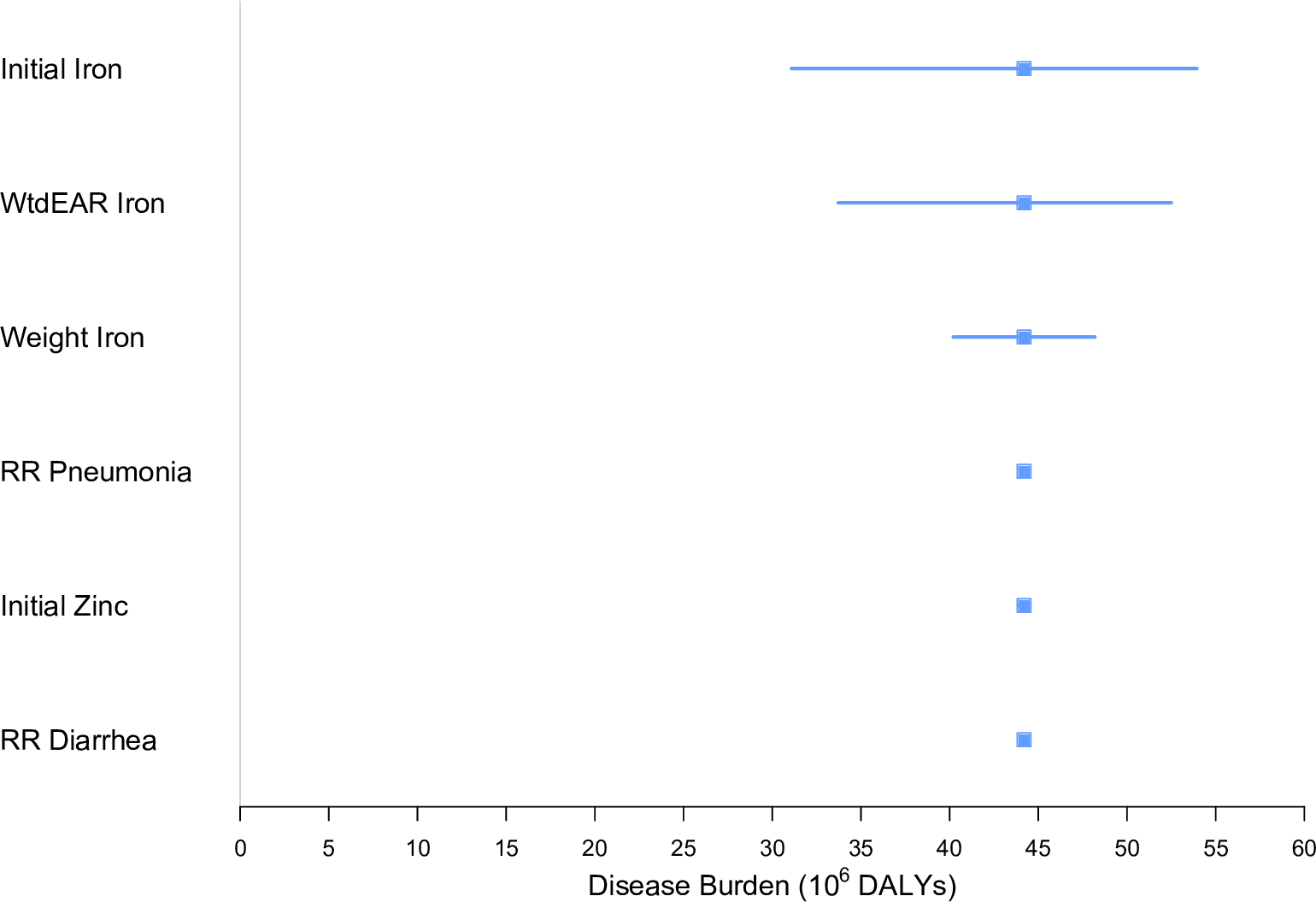

Supplement: S5 Fig — One-way sensitivity analysis was performed across all model inputs. For each country, inputs were varied by plus and minus 10%, and the microsimulation was run 1,000 times. The 6 most influential inputs are shown for the South-East Asia Region. (TIF) [file pmed.1002586.s005.tif]

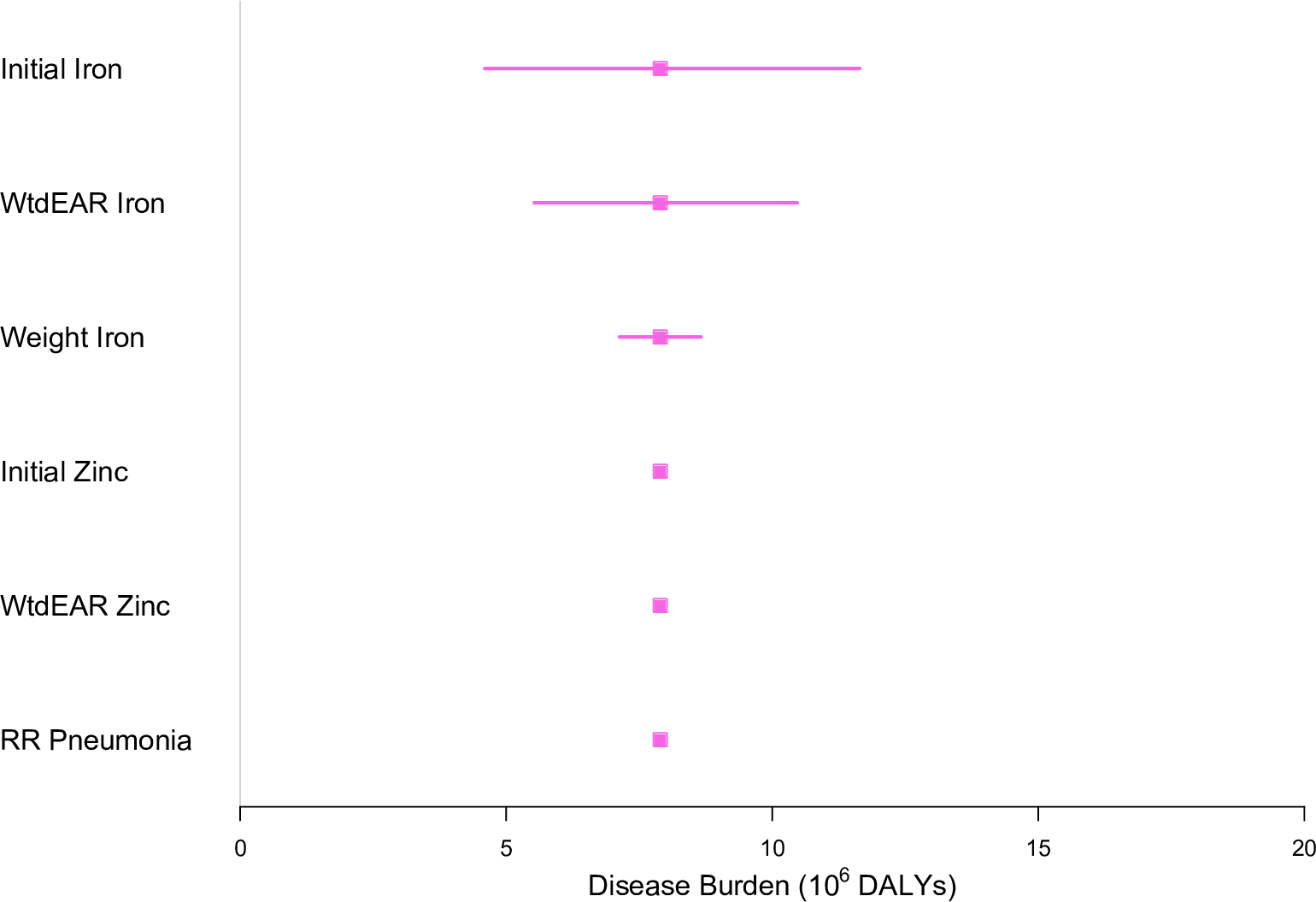

Supplement: S6 Fig — One-way sensitivity analysis was performed across all model inputs. For each country, inputs were varied by plus and minus 10%, and the microsimulation was run 1,000 times. The 6 most influential inputs are shown for the European Region. (TIF) [file pmed.1002586.s006.tif]

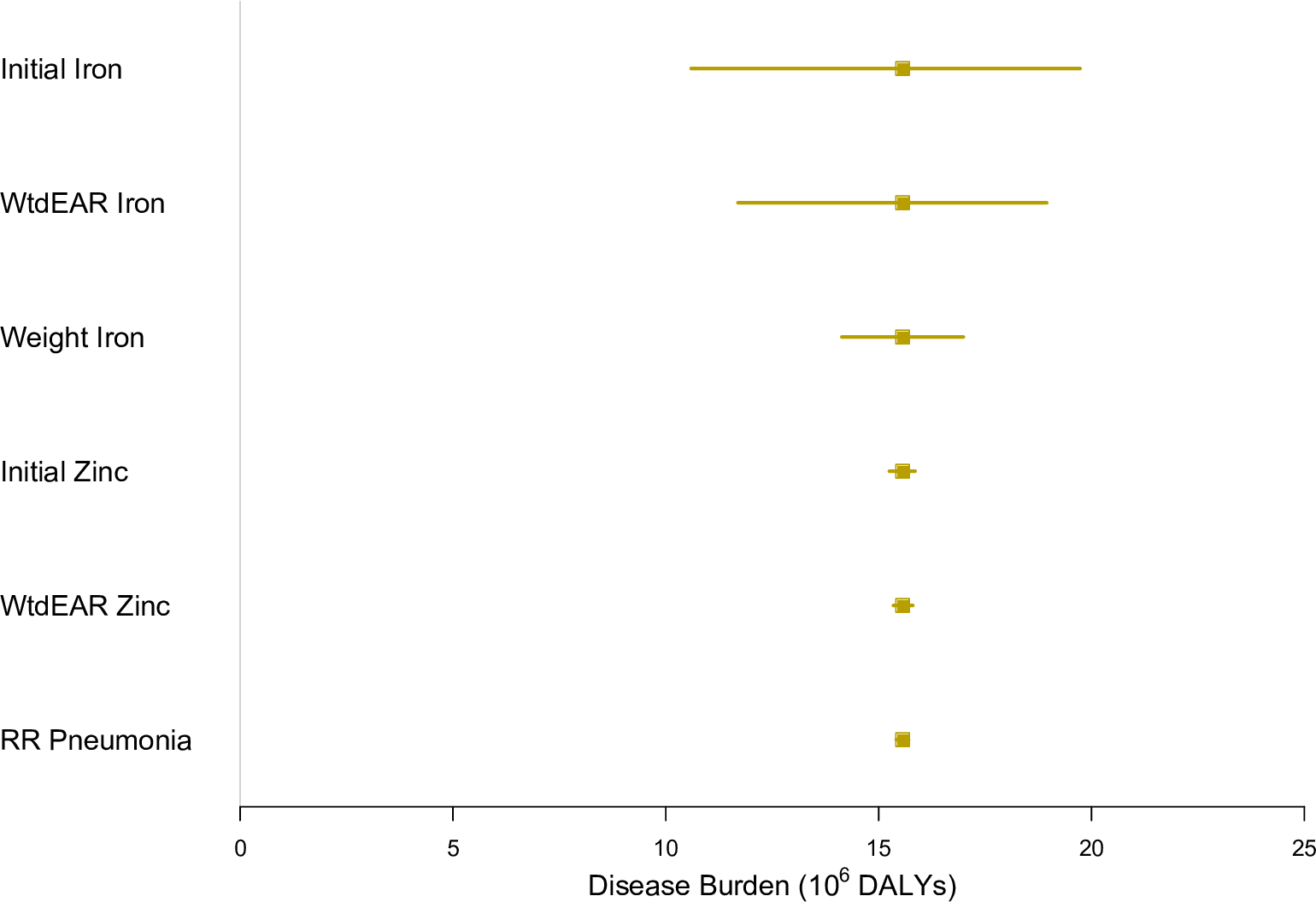

Supplement: S7 Fig — One-way sensitivity analysis was performed across all model inputs. For each country, inputs were varied by plus and minus 10%, and the microsimulation was run 1,000 times. The 6 most influential inputs are shown for the Eastern Mediterranean Region. (TIF) [file pmed.1002586.s007.tif]

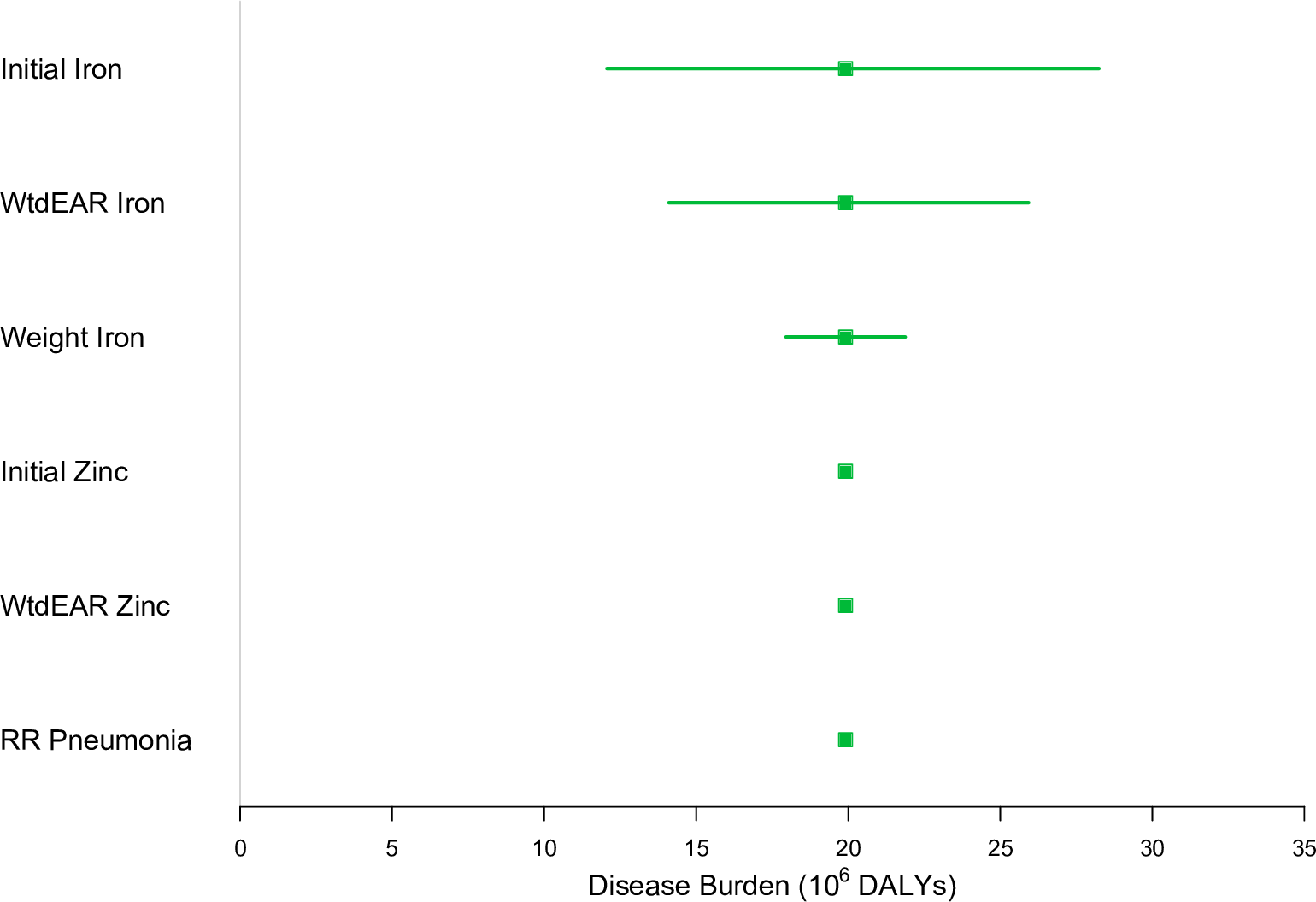

Supplement: S8 Fig — One-way sensitivity analysis was performed across all model inputs. For each country, inputs were varied by plus and minus 10%, and the microsimulation was run 1,000 times. The 6 most influential inputs are shown for the Western Pacific Region. (TIF) [file pmed.1002586.s008.tif]

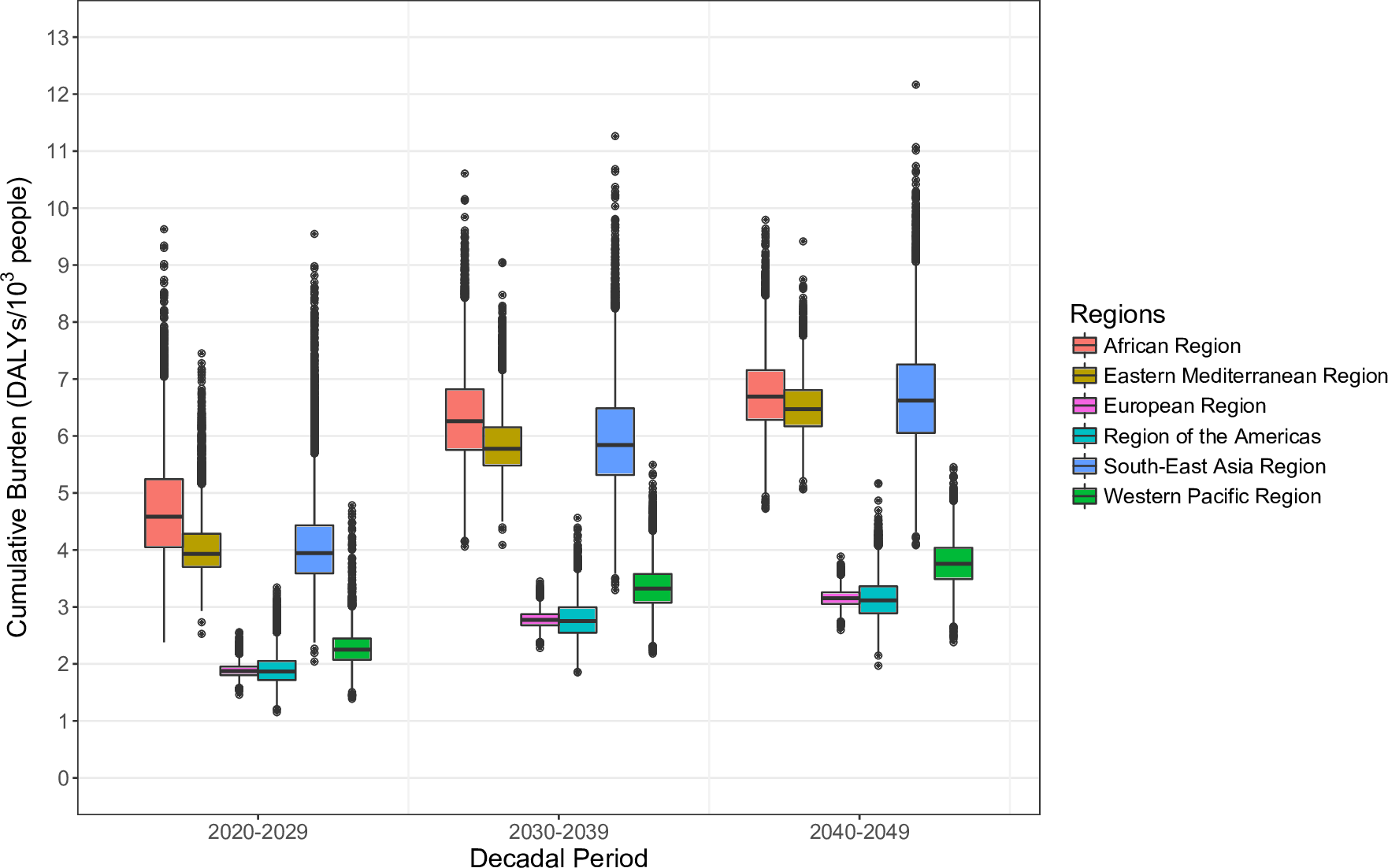

Supplement: S9 Fig — The microsimulation was run 10,000 times for each country. The boxplots show median, interquartile, and outlier cumulative per capita carbon-dioxide-induced DALY burdens by decadal period for the countries in each region. These values reflect the additional zinc and iron deficiency burden of disease due to increasing carbon dioxide, and background disease burdens are subtracted. (TIF) [file pmed.1002586.s009.tif]

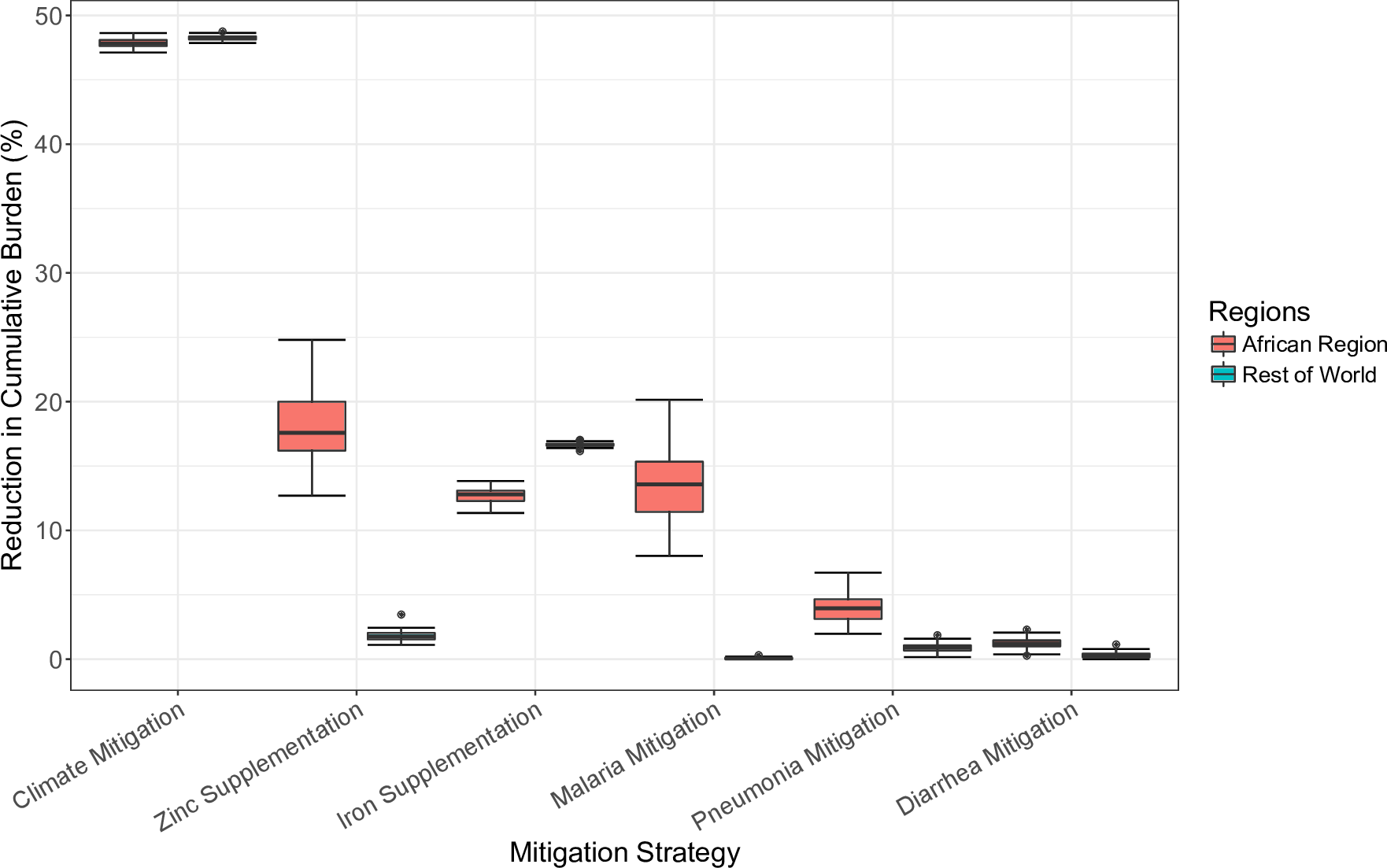

Supplement: S10 Fig — The microsimulation of 1 million people was run 100 times per country per mitigation strategy. The boxplots show median, interquartile, and outlier reductions in cumulative DALY burdens from 2015 to 2050 for countries by region by strategy. (TIF) [file pmed.1002586.s010.tif]
