# Supplementary material for: Anticipated burden and mitigation of carbon-dioxide-induced nutritional deficiencies and related diseases: A simulation modeling study
Source: PLoS Med. 2018 Jul 3;15(7):e1002586. doi: 10.1371/journal.pmed.1002586 (PMC6029750; doi:10.1371/journal.pmed.1002586)
Supplement: S1 Table — Results are shown as percent changes from literature values to model results. Iron supply could not be validated at the regional level as literature values were not available. (DOCX) [file pmed.1002586.s011.docx]

| **Region** | **Zinc Supply (%)** | **Zinc Deficiency (%)** | **Iron Deficiency (%)** |
| --- | --- | --- | --- |
| Global | -0.8 | 3.3 | 0.6 |
| African Region | 0.4 | -0.6 | 1.1 |
| Region of the Americas | -2.4 | 7.7 | 1.5 |
| South-East Asia Region | -0.7 | 4.1 | 0.1 |
| European Region | -3.0 | 18.1 | 1.3 |
| Eastern Mediterranean Region | 0.1 | -12.0 | 0.3 |
| Western Pacific Region | 0.6 | 8.1 | 0.3 |
