# Supplementary material for: Anticipated burden and mitigation of carbon-dioxide-induced nutritional deficiencies and related diseases: A simulation modeling study
Source: PLoS Med. 2018 Jul 3;15(7):e1002586. doi: 10.1371/journal.pmed.1002586 (PMC6029750; doi:10.1371/journal.pmed.1002586)
Supplement: S2 Table — Results are shown as percent changes from literature values to model results. (DOCX) [file pmed.1002586.s012.docx]

| **Region** | **Malaria (%)** | **Pneumonia (%)** | **Diarrhea (%)** | **Anemia (%)** |
| --- | --- | --- | --- | --- |
| Global | 2.4 | -0.3 | 1.4 | -0.2 |
| African Region | 2.4 | -2.1 | 0.8 | 0.3 |
| Region of the Americas | -4.8 | -3.7 | -2.8 | 1.2 |
| South-East Asia Region | 1.2 | -0.1 | 0.0 | -0.7 |
| European Region | -2.8 | 1.7 | -1.1 | 0.5 |
| Eastern Mediterranean Region | 3.2 | 8.3 | 6.6 | -0.4 |
| Western Pacific Region | -0.7 | -5.7 | -2.0 | -0.4 |
