# Supplementary material for: Anticipated burden and mitigation of carbon-dioxide-induced nutritional deficiencies and related diseases: A simulation modeling study
Source: PLoS Med. 2018 Jul 3;15(7):e1002586. doi: 10.1371/journal.pmed.1002586 (PMC6029750; doi:10.1371/journal.pmed.1002586)
Supplement: S3 Table — Results are shown as percent changes from literature values to model results. (DOCX) [file pmed.1002586.s013.docx]

| **Region** | **Malaria (%)** | **Pneumonia (%)** | **Diarrhea (%)** | **Anemia (%)** |
| --- | --- | --- | --- | --- |
| Global | -0.7 | -1.3 | -2.0 | 2.7 |
| African Region | -0.6 | -3.6 | -2.5 | 6.2 |
| Region of the Americas | -3.8 | -6.3 | -4.4 | 1.6 |
| South-East Asia Region | -5.4 | -2.8 | -3.0 | 2.2 |
| European Region | -6.8 | -2.5 | -3.2 | -0.8 |
| Eastern Mediterranean Region | -0.4 | 8.5 | 1.7 | 4.9 |
| Western Pacific Region | -2.9 | 4.8 | -3.0 | -0.5 |
