# Supplementary material for: Anticipated burden and mitigation of carbon-dioxide-induced nutritional deficiencies and related diseases: A simulation modeling study
Source: PLoS Med. 2018 Jul 3;15(7):e1002586. doi: 10.1371/journal.pmed.1002586 (PMC6029750; doi:10.1371/journal.pmed.1002586)
Supplement: S4 Table — The microsimulation of 10,000 people was run 10,000 times for each country. Projected raw and per capita DALY burdens due to carbon-dioxide-induced declines in the zinc and iron concentrations of crops from 2015 to 2050 are shown. Micronutrient supplies and percent deficiencies in 2015 are also shown. (DOCX) [file pmed.1002586.s014.docx]

| **Country** | **Burden**  **(10^6^ DALYs)** | **Burden per capita**  **(DALYs/10^3^ ppl)** | **Zn Supply**  **(mg/capita/day)** | **Iron Supply**  **(mg/capita/day)** | **Zinc Deficiency (%)** | **Iron Deficiency (%)** |
| --- | --- | --- | --- | --- | --- | --- |
| Bolivia | 0.2 | 16.8 | 14.2 | 17.6 | 13.8 | 18.3 |
| Ecuador | 0.3 | 14.3 | 13.1 | 15.8 | 19.4 | 18 |
| Peru | 0.5 | 12 | 12.7 | 25.9 | 22.7 | 15.8 |
| Australia | 0.2 | 7.8 | 19.8 | 17.3 | 2.7 | 8.9 |
| New Zealand | 0 | 6.9 | 21.1 | 18.1 | 1.9 | 7.9 |
| Cuba | 0.1 | 10.4 | 15.9 | 27.9 | 7.8 | 18.5 |
| Dominican Republic | 0.2 | 14.5 | 10.4 | 17.9 | 48.5 | 18.9 |
| Haiti | 0.3 | 23.7 | 9.3 | 18.3 | 66.9 | 24.4 |
| Jamaica | 0 | 15.1 | 13 | 21 | 20.4 | 18.9 |
| Trinidad and Tobago | 0 | 12 | 14.2 | 20 | 13.2 | 18.6 |
| Armenia | 0 | 10.4 | 19.4 | 19.2 | 3 | 12.9 |
| Azerbaijan | 0.1 | 13.1 | 23 | 24.3 | 1.4 | 16 |
| Georgia | 0 | 10.2 | 17.8 | 19.9 | 4.1 | 14.9 |
| Kazakhstan | 0.2 | 8.8 | 21.2 | 18.7 | 1.9 | 14.8 |
| Kyrgyzstan | 0.1 | 10.6 | 21.2 | 20.8 | 2 | 15.6 |
| Mongolia | 0 | 7.7 | 24.3 | 17.8 | 1.1 | 9.9 |
| Tajikistan | 0.2 | 16.9 | 14.4 | 17.1 | 13.5 | 17.4 |
| Turkmenistan | 0.1 | 12.1 | 27.7 | 25.1 | 0.6 | 15.2 |
| Uzbekistan | 0.4 | 10.6 | 22.9 | 22.1 | 1.4 | 19.2 |
| Albania | 0 | 15.7 | 20.7 | 22.6 | 2.4 | 14.6 |
| Bosnia and Herzegovina | 0 | 12.8 | 19.8 | 25.5 | 2.7 | 10.7 |
| Bulgaria | 0.1 | 10.5 | 16 | 17.9 | 7.1 | 10.6 |
| Croatia | 0 | 9.7 | 16.1 | 17.1 | 6.9 | 10.6 |
| Czech Republic | 0.1 | 9.2 | 15.1 | 16.9 | 9.5 | 10.6 |
| Hungary | 0.1 | 8.7 | 13.3 | 14.7 | 17.1 | 10.7 |
| Macedonia | 0 | 12.3 | 16 | 20.5 | 7.6 | 10.2 |
| Poland | 0.3 | 9.1 | 17.6 | 19.4 | 4.5 | 10.6 |
| Romania | 0.2 | 11.8 | 20 | 22.7 | 2.5 | 11 |
| Serbia | 0.1 | 10.9 | 17.2 | 18.1 | 5.1 | 9.6 |
| Slovakia | 0 | 9.7 | 14.1 | 16.2 | 13 | 10.6 |
| Slovenia | 0 | 9.8 | 18.2 | 19.1 | 3.8 | 10.5 |
| Colombia | 0.3 | 6.1 | 13.1 | 18.1 | 19.4 | 20.7 |
| Costa Rica | 0 | 7.7 | 12.8 | 20.4 | 22.1 | 12.7 |
| El Salvador | 0.1 | 18.1 | 13.4 | 17.7 | 18.2 | 16.6 |
| Guatemala | 0.6 | 24.1 | 13.4 | 17.4 | 17.9 | 17.6 |
| Honduras | 0.2 | 18.7 | 13.4 | 16.3 | 18.4 | 13 |
| Mexico | 1.8 | 12.1 | 17.3 | 18.4 | 5.1 | 11.9 |
| Nicaragua | 0.1 | 11.9 | 13.1 | 20.8 | 20.1 | 14.4 |
| Panama | 0.1 | 10.4 | 12.8 | 20.9 | 21.5 | 15.2 |
| Venezuela | 0.2 | 5.4 | 15 | 18 | 10.6 | 13.5 |
| Angola | 0.9 | 18.9 | 11.4 | 14.2 | 34.5 | 20.6 |
| Central African Republic | 0.2 | 22.8 | 11.8 | 13.9 | 31.1 | 25.4 |
| Congo | 0.1 | 18.6 | 8.8 | 12.6 | 74.7 | 23.8 |
| Gabon | 0.1 | 22.6 | 13 | 19.3 | 20.9 | 18.2 |
| China | 12.9 | 9.4 | 19.2 | 31.5 | 3.2 | 12.4 |
| North Korea | 0.4 | 15.9 | 11.3 | 24.6 | 36.3 | 16.5 |
| Cambodia | 0.5 | 22.3 | 10.6 | 32.5 | 45.7 | 25.7 |
| Indonesia | 4.7 | 15.4 | 11.5 | 30.1 | 33.3 | 18.5 |
| Laos | 0.2 | 22.2 | 11 | 31.5 | 41 | 21.6 |
| Malaysia | 0.3 | 7.6 | 13.4 | 25.7 | 17.8 | 13.4 |
| Mauritius | 0 | 8.3 | 16.6 | 26.1 | 6.5 | 10 |
| Myanmar | 0.7 | 11 | 11.8 | 31.9 | 30.3 | 19.3 |
| Philippines | 2.2 | 16.5 | 11.3 | 26.4 | 36.6 | 18.1 |
| Sri Lanka | 0.3 | 15.1 | 10.7 | 29.8 | 42.9 | 19.7 |
| Thailand | 0.3 | 5.3 | 10.6 | 28.5 | 44.1 | 12.4 |
| Timor-Leste | 0 | 21 | 11.6 | 20.8 | 35.3 | 24.5 |
| Vietnam | 1.6 | 14.6 | 14 | 32.7 | 14.6 | 14.8 |
| Argentina | 0.4 | 7.1 | 26.1 | 19.4 | 0.7 | 9.9 |
| Chile | 0.1 | 7 | 19 | 19.9 | 3.3 | 8.6 |
| Uruguay | 0 | 9 | 21.1 | 20.7 | 2 | 9.8 |
| Botswana | 0 | 12.7 | 12.1 | 15.4 | 28.9 | 17.5 |
| Lesotho | 0.1 | 27 | 18.7 | 21.4 | 3.8 | 17 |
| Namibia | 0 | 13.7 | 11.9 | 13.8 | 30.6 | 20.8 |
| South Africa | 0.8 | 12.9 | 19 | 20.3 | 3.3 | 17 |
| Swaziland | 0 | 21.3 | 13.6 | 15.8 | 17.1 | 16.7 |
| Zimbabwe | 0.7 | 29.8 | 12.4 | 14.3 | 26 | 21.2 |
| Brazil | 1.1 | 4.7 | 18.9 | 22.5 | 3.4 | 13.9 |
| Paraguay | 0.1 | 6.8 | 13.5 | 13.8 | 17.7 | 14.9 |
| Austria | 0.1 | 8.2 | 17.4 | 17.8 | 4.9 | 8.4 |
| Belgium | 0.1 | 7.8 | 18.2 | 19.9 | 3.9 | 8.3 |
| Cyprus | 0 | 5.6 | 14.7 | 15.5 | 11.6 | 8.1 |
| Denmark | 0.1 | 10.5 | 18.1 | 18 | 4.1 | 7.4 |
| Finland | 0 | 6.4 | 20.1 | 17.5 | 2.4 | 8.4 |
| France | 0.5 | 7.6 | 20.6 | 19 | 2.1 | 8.4 |
| Germany | 0.6 | 7.7 | 16.7 | 17 | 5.9 | 8.2 |
| Greece | 0.1 | 6 | 20.2 | 21.3 | 2.3 | 8.3 |
| Ireland | 0 | 9.4 | 21.1 | 20.3 | 1.9 | 8.4 |
| Israel | 0.1 | 11.3 | 23.1 | 25.3 | 1.3 | 8.3 |
| Italy | 0.4 | 6.7 | 21.7 | 21.4 | 1.6 | 8.1 |
| Netherlands | 0.1 | 8.4 | 17.7 | 16.6 | 4.5 | 8.5 |
| Norway | 0.1 | 10.1 | 19 | 18.8 | 3.2 | 8 |
| Portugal | 0.1 | 8.3 | 19 | 20.3 | 3 | 8.3 |
| Spain | 0.4 | 8.9 | 16.3 | 17.8 | 6.4 | 7.9 |
| Sweden | 0.1 | 8.5 | 17 | 16 | 5.5 | 8.4 |
| Switzerland | 0.1 | 8.3 | 17.5 | 16.4 | 4.8 | 8.4 |
| United Kingdom | 0.5 | 7.4 | 18.7 | 18.9 | 3.4 | 7.5 |
| Benin | 0.4 | 23.2 | 11 | 22.9 | 40.6 | 27.6 |
| Burkina Faso | 0.6 | 19.4 | 16 | 24.2 | 8.7 | 29.3 |
| Cameroon | 0.9 | 23.5 | 12.6 | 22.4 | 23.9 | 21.1 |
| Chad | 0.7 | 29.5 | 12.4 | 18.6 | 27.1 | 25.8 |
| Côte d’Ivoire | 1 | 27.6 | 10.1 | 21.2 | 54.8 | 23.1 |
| Ghana | 0.8 | 20.5 | 10.4 | 20.3 | 48.5 | 23.9 |
| Guinea | 0.7 | 31.7 | 9.9 | 23.9 | 57.5 | 26 |
| Mali | 0.9 | 26.6 | 17 | 27.3 | 6.5 | 30.5 |
| Mauritania | 0.2 | 28.2 | 16.1 | 22.3 | 7.6 | 24.7 |
| Niger | 1.1 | 23.2 | 17.5 | 29 | 7.1 | 28.1 |
| Nigeria | 7.4 | 24.8 | 12.2 | 22.6 | 28.2 | 26.1 |
| Senegal | 0.9 | 32.5 | 11.8 | 23 | 31.3 | 26.3 |
| Belarus | 0 | 5.8 | 18.3 | 18.7 | 3.6 | 12.8 |
| Estonia | 0 | 7.2 | 18 | 19.1 | 3.8 | 12.7 |
| Latvia | 0 | 7.2 | 15.9 | 16.9 | 6.8 | 12.8 |
| Lithuania | 0 | 6.5 | 18.4 | 20.8 | 3.5 | 12.7 |
| Moldova | 0 | 11.9 | 14.7 | 15.1 | 10.8 | 14.4 |
| Russia | 0.9 | 6.7 | 19.7 | 20.5 | 2.5 | 12.7 |
| Ukraine | 0.2 | 4 | 16.9 | 19.2 | 5.2 | 12.7 |
| Ethiopia | 2.3 | 15.2 | 13.5 | 19.2 | 18 | 18.5 |
| Kenya | 1 | 13.9 | 14.1 | 17.4 | 14.2 | 19.9 |
| Madagascar | 0.9 | 21.6 | 9.3 | 22.7 | 67.4 | 24.2 |
| Malawi | 0.5 | 16.1 | 13.9 | 19.4 | 15 | 23.1 |
| Mozambique | 1.1 | 22.9 | 9.9 | 15.4 | 55.9 | 23.4 |
| Rwanda | 0.2 | 9.5 | 9.3 | 19.2 | 67.4 | 18.1 |
| Tanzania | 1.7 | 17.5 | 11.5 | 18.1 | 34 | 28.5 |
| Uganda | 1.2 | 16.2 | 10.5 | 16 | 47.7 | 21.6 |
| Zambia | 0.5 | 18.2 | 11.7 | 13.4 | 31.9 | 19.8 |
| Japan | 1.4 | 12.7 | 14.2 | 24.3 | 12.4 | 9 |
| South Korea | 0.8 | 15.7 | 18 | 31.7 | 4.3 | 9.3 |
| Canada | 0.2 | 5.7 | 17.8 | 20 | 4.4 | 8.3 |
| United States | 2.9 | 8.4 | 18.2 | 17.5 | 4 | 7.7 |
| Afghanistan | 2.3 | 47.5 | 16.9 | 21.2 | 6.2 | 17.2 |
| Algeria | 0.8 | 15 | 20.5 | 25.1 | 2.3 | 15.6 |
| Egypt | 2.2 | 17.6 | 23 | 33.4 | 1.4 | 19.8 |
| Iran | 0.9 | 9.8 | 18.7 | 27.9 | 3.6 | 15.8 |
| Iraq | 1.3 | 21.2 | 14.7 | 21.9 | 11.8 | 17.8 |
| Jordan | 0.2 | 15.9 | 18.2 | 24.2 | 4.2 | 15.4 |
| Kuwait | 0.1 | 13.6 | 20.6 | 29.5 | 2.7 | 13.4 |
| Lebanon | 0.1 | 13.4 | 19.1 | 23.9 | 3.6 | 14.8 |
| Morocco | 0.7 | 17.3 | 21.7 | 27.3 | 1.8 | 17.2 |
| Saudi Arabia | 0.5 | 13 | 17.1 | 24.6 | 6.1 | 15.5 |
| Tunisia | 0.2 | 13.8 | 22.1 | 28.3 | 1.6 | 13.7 |
| Turkey | 1.4 | 15.2 | 21.7 | 29.2 | 1.7 | 15.1 |
| United Arab Emirates | 0.1 | 10.6 | 18.8 | 31.1 | 4.8 | 14.2 |
| Yemen | 1.1 | 28.1 | 14.3 | 19.2 | 14.1 | 27.5 |
| Bangladesh | 4.5 | 22.8 | 10.2 | 35.2 | 53.2 | 21.3 |
| India | 32.4 | 20.5 | 12.1 | 26.2 | 28.8 | 23 |
| Nepal | 0.7 | 19.3 | 14.6 | 30.1 | 12 | 23.2 |
| Pakistan | 5.1 | 19.4 | 14.7 | 18 | 12.4 | 19.2 |
| Sudan | 0.6 | 10 | 14.9 | 17.4 | 12.5 | 23.9 |
