# Supplementary material for: Anticipated burden and mitigation of carbon-dioxide-induced nutritional deficiencies and related diseases: A simulation modeling study
Source: PLoS Med. 2018 Jul 3;15(7):e1002586. doi: 10.1371/journal.pmed.1002586 (PMC6029750; doi:10.1371/journal.pmed.1002586)
Supplement: S5 Table — (DOCX) [file pmed.1002586.s015.docx]

| **Parameter** | **Probability Distributions** |
| --- | --- |
| Zinc and iron concentrations | Gamma |
| Initial zinc and iron | Normal |
| Slopes of zinc and iron concentrations | Normal |
| WtdEAR iron | Gamma |
| Disease prevalence rates | Beta |
| Relative risks of diseases given zinc deficiency | Gamma |
| Disease mortalities | Gamma |
