# Supplementary material for: Anticipated burden and mitigation of carbon-dioxide-induced nutritional deficiencies and related diseases: A simulation modeling study
Source: PLoS Med. 2018 Jul 3;15(7):e1002586. doi: 10.1371/journal.pmed.1002586 (PMC6029750; doi:10.1371/journal.pmed.1002586)
Supplement: S6 Table — (DOCX) [file pmed.1002586.s016.docx]

| **Region** | **Burden (10^6^ DALYs)** | **95% Credible Interval (10^6^ DALYs)** |
| --- | --- | --- |
| Global | 125.8 | (113.6, 138.9) |
| African Region | 28.5 | (24.2, 33.4) |
| Region of the Americas | 9.8 | (7.9, 12.2) |
| South-East Asia Region | 44.0 | (34.3, 55.1) |
| European Region | 7.9 | (7.2, 8.7) |
| Eastern Mediterranean Region | 15.5 | (13.6, 17.7) |
| Western Pacific Region | 20.0 | (15.9, 24.5) |
