# Supplementary material for: Anticipated burden and mitigation of carbon-dioxide-induced nutritional deficiencies and related diseases: A simulation modeling study
Source: PLoS Med. 2018 Jul 3;15(7):e1002586. doi: 10.1371/journal.pmed.1002586 (PMC6029750; doi:10.1371/journal.pmed.1002586)
Supplement: S7 Table — Iron consumption distributions were assumed to be lognormal with 40% CVs. The model was run 10,000 times with 10,000 people while sampling from distributions reflecting uncertainty in inputs. (DOCX) [file pmed.1002586.s017.docx]

| **Region** | **Burden (10^6^ DALYs)** | **95% Credible Interval (10^6^ DALYs)** |
| --- | --- | --- |
| Global | 122.2 | (111.1, 134.3) |
| African Region | 26.4 | (22.5, 31.0) |
| Region of the Americas | 10.6 | (8.8, 12.5) |
| South-East Asia Region | 40.5 | (31.6, 50.5) |
| European Region | 8.7 | (8.0, 9.4) |
| Eastern Mediterranean Region | 14.9 | (13.0, 17.0) |
| Western Pacific Region | 21.2 | (17.2, 25.5) |
