# Supplementary material for: Anticipated burden and mitigation of carbon-dioxide-induced nutritional deficiencies and related diseases: A simulation modeling study
Source: PLoS Med. 2018 Jul 3;15(7):e1002586. doi: 10.1371/journal.pmed.1002586 (PMC6029750; doi:10.1371/journal.pmed.1002586)
Supplement: S8 Table — Malaria, pneumonia, and diarrhea prevalence rates in 2015 were assumed to continue over the model period. The model was run 10,000 times with 10,000 people while sampling from distributions reflecting uncertainty in inputs. (DOCX) [file pmed.1002586.s018.docx]

| **Region** | **Burden (10^6^ DALYs)** | **95% Credible Interval (10^6^ DALYs)** |
| --- | --- | --- |
| Global | 131.4 | (118.7, 145.3) |
| African Region | 31.7 | (27.0, 37.1) |
| Region of the Americas | 9.9 | (8.1, 12.1) |
| South-East Asia Region | 45.7 | (35.4, 57.8) |
| European Region | 8.0 | (7.3, 8.7) |
| Eastern Mediterranean Region | 16.0 | (13.9, 18.4) |
| Western Pacific Region | 20.0 | (16.0, 24.7) |
