# Supplementary material for: Anticipated burden and mitigation of carbon-dioxide-induced nutritional deficiencies and related diseases: A simulation modeling study
Source: PLoS Med. 2018 Jul 3;15(7):e1002586. doi: 10.1371/journal.pmed.1002586 (PMC6029750; doi:10.1371/journal.pmed.1002586)
Supplement: S9 Table — The microsimulation of 1 million people was run 100 times per country per mitigation strategy. Six mitigation strategies were considered. They included climate mitigation per the Paris Agreement to keep global temperatures below 2°C of pre-industrial levels; daily zinc supplementation for 80% of children under 5 years of age; weekly iron supplementation for 80% of women; and malaria, pneumonia, and diarrhea mitigation programs previously described in the literature adopted at 80% coverage rates. The mean percent reductions in cumulative DALY burdens due to carbon-dioxide-induced declines in zinc and iron concentrations of crops from 2015 to 2050 are shown. (DOCX) [file pmed.1002586.s019.docx]

| **Country** | **Climate (%)** | **Zinc (%)** | **Iron (%)** | **Malaria (%)** | **Pneumonia (%)** | **Diarrhea (%)** |
| --- | --- | --- | --- | --- | --- | --- |
| Bolivia | 48 | 1 | 17 | 0 | 0 | 0 |
| Ecuador | 48 | 2 | 17 | 0 | 1 | 0 |
| Peru | 48 | 1 | 17 | 0 | 0 | 0 |
| Australia | 49 | 0 | 17 | 0 | 0 | 0 |
| New Zealand | 49 | 0 | 18 | 0 | 0 | 0 |
| Cuba | 48 | 0 | 18 | 0 | 0 | 0 |
| Dominican Republic | 48 | 1 | 17 | 0 | 1 | 0 |
| Haiti | 48 | 2 | 17 | 0 | 1 | 0 |
| Jamaica | 48 | 1 | 18 | 0 | 0 | 0 |
| Trinidad and Tobago | 48 | 1 | 18 | 0 | 0 | 0 |
| Armenia | 49 | 0 | 19 | 0 | 0 | 0 |
| Azerbaijan | 48 | 1 | 17 | 0 | 0 | 0 |
| Georgia | 49 | 0 | 19 | 0 | 0 | 0 |
| Kazakhstan | 48 | 0 | 17 | 0 | 0 | 0 |
| Kyrgyzstan | 48 | 1 | 17 | 0 | 0 | 0 |
| Mongolia | 48 | 0 | 17 | 0 | 0 | 0 |
| Tajikistan | 48 | 5 | 16 | 0 | 3 | 1 |
| Turkmenistan | 48 | 1 | 17 | 0 | 0 | 0 |
| Uzbekistan | 48 | 2 | 17 | 0 | 1 | 0 |
| Albania | 48 | 0 | 18 | 0 | 0 | 0 |
| Bosnia and Herzegovina | 49 | 0 | 18 | 0 | 0 | 0 |
| Bulgaria | 49 | 0 | 19 | 0 | 0 | 0 |
| Croatia | 49 | 0 | 18 | 0 | 0 | 0 |
| Czech Republic | 49 | 0 | 18 | 0 | 0 | 0 |
| Hungary | 48 | 0 | 18 | 0 | 0 | 0 |
| Macedonia | 49 | 0 | 18 | 0 | 0 | 0 |
| Poland | 48 | 0 | 18 | 0 | 0 | 0 |
| Romania | 49 | 0 | 18 | 0 | 0 | 0 |
| Serbia | 49 | 0 | 18 | 0 | 0 | 0 |
| Slovakia | 49 | 0 | 18 | 0 | 0 | 0 |
| Slovenia | 49 | 0 | 18 | 0 | 0 | 0 |
| Colombia | 48 | 1 | 18 | 0 | 1 | 0 |
| Costa Rica | 49 | 1 | 17 | 0 | 0 | 0 |
| El Salvador | 48 | 0 | 18 | 0 | 0 | 0 |
| Guatemala | 48 | 2 | 17 | 0 | 1 | 0 |
| Honduras | 49 | 1 | 17 | 0 | 0 | 0 |
| Mexico | 49 | 0 | 17 | 0 | 0 | 0 |
| Nicaragua | 48 | 2 | 17 | 0 | 1 | 0 |
| Panama | 48 | 2 | 17 | 0 | 1 | 0 |
| Venezuela | 48 | 1 | 17 | 0 | 0 | 0 |
| Angola | 48 | 20 | 11 | 11 | 6 | 2 |
| Central African Republic | 48 | 16 | 13 | 6 | 6 | 2 |
| Congo | 48 | 9 | 14 | 3 | 4 | 1 |
| Gabon | 48 | 8 | 14 | 3 | 3 | 0 |
| China | 49 | 0 | 17 | 0 | 0 | 0 |
| North Korea | 48 | 1 | 18 | 0 | 0 | 0 |
| Cambodia | 48 | 2 | 17 | 0 | 1 | 0 |
| Indonesia | 48 | 2 | 17 | 0 | 1 | 0 |
| Laos | 48 | 3 | 16 | 0 | 2 | 0 |
| Malaysia | 49 | 0 | 17 | 0 | 0 | 0 |
| Mauritius | 48 | 3 | 17 | 0 | 1 | 0 |
| Myanmar | 48 | 2 | 17 | 0 | 1 | 0 |
| Philippines | 48 | 1 | 17 | 0 | 1 | 0 |
| Sri Lanka | 48 | 0 | 18 | 0 | 0 | 0 |
| Thailand | 49 | 1 | 18 | 0 | 0 | 0 |
| Timor-Leste | 48 | 2 | 16 | 0 | 1 | 0 |
| Vietnam | 48 | 3 | 17 | 0 | 2 | 0 |
| Argentina | 48 | 0 | 18 | 0 | 0 | 0 |
| Chile | 49 | 0 | 18 | 0 | 0 | 0 |
| Uruguay | 49 | 0 | 18 | 0 | 0 | 0 |
| Botswana | 48 | 3 | 18 | 1 | 1 | 1 |
| Lesotho | 48 | 4 | 20 | 0 | 2 | 1 |
| Namibia | 48 | 4 | 16 | 2 | 1 | 1 |
| South Africa | 48 | 1 | 17 | 0 | 0 | 0 |
| Swaziland | 48 | 11 | 17 | 1 | 5 | 3 |
| Zimbabwe | 48 | 15 | 15 | 1 | 3 | 11 |
| Brazil | 48 | 0 | 18 | 0 | 0 | 0 |
| Paraguay | 48 | 1 | 17 | 0 | 1 | 0 |
| Austria | 49 | 0 | 18 | 0 | 0 | 0 |
| Belgium | 49 | 0 | 18 | 0 | 0 | 0 |
| Cyprus | 49 | 0 | 18 | 0 | 0 | 0 |
| Denmark | 49 | 0 | 18 | 0 | 0 | 0 |
| Finland | 49 | 0 | 18 | 0 | 0 | 0 |
| France | 49 | 0 | 18 | 0 | 0 | 0 |
| Germany | 49 | 0 | 18 | 0 | 0 | 0 |
| Greece | 49 | 0 | 18 | 0 | 0 | 0 |
| Ireland | 49 | 0 | 17 | 0 | 0 | 0 |
| Israel | 49 | 0 | 17 | 0 | 0 | 0 |
| Italy | 49 | 0 | 19 | 0 | 0 | 0 |
| Netherlands | 49 | 0 | 18 | 0 | 0 | 0 |
| Norway | 49 | 0 | 18 | 0 | 0 | 0 |
| Portugal | 49 | 0 | 19 | 0 | 0 | 0 |
| Spain | 49 | 0 | 18 | 0 | 0 | 0 |
| Sweden | 49 | 0 | 18 | 0 | 0 | 0 |
| Switzerland | 49 | 0 | 18 | 0 | 0 | 0 |
| United Kingdom | 49 | 0 | 18 | 0 | 0 | 0 |
| Benin | 48 | 10 | 14 | 9 | 1 | 0 |
| Burkina Faso | 48 | 15 | 13 | 13 | 2 | 1 |
| Cameroon | 48 | 21 | 12 | 13 | 7 | 1 |
| Chad | 48 | 25 | 10 | 10 | 8 | 5 |
| Côte d’Ivoire | 48 | 18 | 12 | 11 | 5 | 1 |
| Ghana | 48 | 12 | 14 | 10 | 3 | 0 |
| Guinea | 48 | 15 | 13 | 12 | 3 | 0 |
| Mali | 48 | 15 | 14 | 15 | 1 | 0 |
| Mauritania | 48 | 6 | 15 | 4 | 1 | 0 |
| Niger | 48 | 16 | 12 | 17 | 1 | 0 |
| Nigeria | 48 | 31 | 10 | 29 | 5 | 1 |
| Senegal | 48 | 5 | 15 | 3 | 1 | 0 |
| Belarus | 48 | 0 | 19 | 0 | 0 | 0 |
| Estonia | 48 | 0 | 18 | 0 | 0 | 0 |
| Latvia | 48 | 0 | 19 | 0 | 0 | 0 |
| Lithuania | 48 | 0 | 19 | 0 | 0 | 0 |
| Moldova | 48 | 0 | 18 | 0 | 0 | 0 |
| Russia | 48 | 0 | 19 | 0 | 0 | 0 |
| Ukraine | 48 | 0 | 19 | 0 | 0 | 0 |
| Ethiopia | 48 | 8 | 15 | 2 | 4 | 1 |
| Kenya | 48 | 10 | 14 | 2 | 5 | 2 |
| Madagascar | 48 | 4 | 15 | 2 | 1 | 1 |
| Malawi | 48 | 13 | 13 | 2 | 7 | 2 |
| Mozambique | 47 | 21 | 13 | 22 | 2 | 1 |
| Rwanda | 48 | 11 | 15 | 5 | 3 | 1 |
| Tanzania | 48 | 15 | 13 | 7 | 6 | 1 |
| Uganda | 47 | 23 | 12 | 18 | 5 | 1 |
| Zambia | 48 | 18 | 13 | 12 | 4 | 2 |
| Japan | 49 | 0 | 18 | 0 | 0 | 0 |
| South Korea | 49 | 0 | 18 | 0 | 0 | 0 |
| Canada | 49 | 0 | 18 | 0 | 0 | 0 |
| United States | 49 | 1 | 18 | 0 | 1 | 0 |
| Afghanistan | 49 | 3 | 17 | 0 | 1 | 0 |
| Algeria | 48 | 10 | 15 | 8 | 2 | 0 |
| Egypt | 48 | 0 | 17 | 0 | 0 | 0 |
| Iran | 48 | 0 | 17 | 0 | 0 | 0 |
| Iraq | 48 | 2 | 16 | 0 | 1 | 0 |
| Jordan | 48 | 1 | 16 | 0 | 0 | 0 |
| Kuwait | 49 | 0 | 16 | 0 | 0 | 0 |
| Lebanon | 48 | 0 | 18 | 0 | 0 | 0 |
| Morocco | 48 | 0 | 17 | 0 | 0 | 0 |
| Saudi Arabia | 48 | 0 | 15 | 0 | 0 | 0 |
| Tunisia | 49 | 0 | 18 | 0 | 0 | 0 |
| Turkey | 49 | 0 | 18 | 0 | 0 | 0 |
| United Arab Emirates | 49 | 0 | 11 | 0 | 0 | 0 |
| Yemen | 48 | 3 | 16 | 2 | 1 | 1 |
| Bangladesh | 48 | 1 | 17 | 0 | 0 | 0 |
| India | 48 | 2 | 16 | 0 | 1 | 0 |
| Nepal | 48 | 1 | 17 | 0 | 1 | 0 |
| Pakistan | 48 | 8 | 14 | 0 | 5 | 2 |
| Sudan | 48 | 3 | 16 | 1 | 1 | 0 |
