# Supplementary material for: Anticipated burden and mitigation of carbon-dioxide-induced nutritional deficiencies and related diseases: A simulation modeling study
Source: PLoS Med. 2018 Jul 3;15(7):e1002586. doi: 10.1371/journal.pmed.1002586 (PMC6029750; doi:10.1371/journal.pmed.1002586)
Supplement: S1 Text — (DOCX) [file pmed.1002586.s020.docx]

**S1 Text: Model inputs**

*Food availability parameters*

Food availability parameters for each country were estimated from the Statistics Division of the Food and Agriculture Organization of the United Nations (FAOSTAT) [1]. The FAOSTAT provides average availability of 87 mutually exclusive and collectively exhaustive food items in each country in kilocalories/capita/day. The FAOSTAT calculates these values from national production, trade, transport losses, storage, non-food uses, and livestock feed, among other factors, but does not account for household waste or inter- and intra-household variation in access to food [1,2].

*Zinc and iron parameters*

Average zinc and iron concentrations of each food were calculated from data from the U.S. Department of Agriculture [3]. The USDA provides average zinc concentration, iron concentration, and energy density of different food items. Food items from the USDA were matched to items in FAOSTAT based on prior mappings [2,4–6]. Average per person zinc and iron availability was calculated by multiplying average availability in grams per person per day of each item by its zinc or iron concentration in milligrams per 100 grams, respectively, and summing.

Zinc consumption distributions for each country were assumed to be normal with 25% coefficients of variation (CVs) based on prior literature [2,4–6]. Iron consumption distributions for each country were also assumed to be normal with 25% CVs [4]. In sensitivity analysis, we used lognormal iron distributions with 40% CVs based on alternative literature [7], which did not materially alter our results (see **S7 Table**).

Zinc and iron requirements for each country were estimated from weighted estimated average requirements (WtdEARs). WtdEARs for zinc for each country were previously calculated by Kumssa et al. [2]. WtdEARs for iron for each country were calculated from iron deficiency anemia (IDA) rates estimated by Kassebaum et al. [8] and the iron distributions noted above*.* There are two potential limitations with this approach. First, the estimated IDA rates “include acute and chronic hemorrhagic states for which supplementation may be helpful, but poor nutritional intake is not the only underlying problem… hookworm, schistosomiasis, upper gastrointestinal bleeding, and gynecologic diseases” may also affect these rates [8]. Second, the EAR cut-point method is not ideal for estimating iron deficiency and IDA among menstruating women as their iron requirements are skewed. Though the model does not specifically consider this group, menstruating women are included in its country-level estimates. However, we know of no validated method for overcoming the above two limitations with available data, and past country-level estimates of the prevalence of iron deficiency have used the EAR cut-point method without further adjustment [4]. In the presence of additional data, an approach that adjusts for these factors might be preferable and considered in future research [9].

*Mortality rates*

Demographic-specific mortality rates and life expectancies were estimated from World Health Organization (WHO) [10]. Disease-specific mortality rates were estimated from the Global Burden of Disease Project (GBD) [11]. General and disease-specific mortality trends over time were also estimated from the GBD [11].

*Disease parameters*

Disease prevalence rates and trends over time were obtained from the GBD [11,12]. Relative risks of morbidity and mortality from malaria, pneumonia, and diarrhea given zinc deficiency in children under 5 years of age were obtained from Caulfield and Black [13]. Disease disability weights were obtained from the GBD and Kassebaum et al. [8,11,12]. In principle, disability weights might vary by age, gender, and personal preference. However, for all diseases considered (malaria, pneumonia, diarrhea illness, and IDA), the GBD reports disability weights that are not stratified by these factors [12]. Thus, in keeping with disability weight methodology set by the GBD, our model did not use stratified disability weights.

*Climate parameters*

Rising atmospheric carbon dioxide concentrations were assumed to alter the micronutrient concentrations of crops from 2015 to 2050 in accordance with the findings of Myers et al. [14]. Myers et al. assumed a rise in atmospheric carbon dioxide concentrations from approximately 400 ppm in 2015 to 550 ppm in 2050. Furthermore, the declines in micronutrient concentrations of particular crops were extrapolated to broad crop categories similar to Myers et al. and Smith et al. [15,16]. See a detailed discussion of the estimation method in Myers et al. [15]. In keeping with their “best-estimate model,” rice was not included in the C_3_ weighted mean. The authors previously found that this exclusion had minimal effect on deficiency estimates.

**S1 Text References**

1. Food and Agriculture Organization of the United Nations. FAOSTAT: food balance sheets. Rome: Food and Agriculture Organization of the United Nations; 2013 [cited 2016 Sep 15]. Available from: http://www.fao.org/faostat/en/#data/FBS.

2. Kumssa DB, Joy EJM, Ander EL, Watts MJ, Young SD, Walker S, et al. Dietary calcium and zinc deficiency risks are decreasing but remain prevalent. Sci Rep. 2015;5:10974.

3. US Department of Agriculture. USDA National Nutrient Database for Standard Reference, Release 28. 2016 [cited 15 Sep 2016]. Available from: https://www.ars.usda.gov/northeast-area/beltsville-md-bhnrc/beltsville-human-nutrition-research-center/nutrient-data-laboratory/docs/usda-national-nutrient-database-for-standard-reference/.

4. Joy EJ, Ander EL, Young SD, Black CR, Watts MJ, Chilimba AD, et al. Dietary mineral supplies in Africa. Physiologia plantarum. 2014;151: 208–229.

5. Wessells KR, Brown KH. Estimating the global prevalence of zinc deficiency: results based on zinc availability in national food supplies and the prevalence of stunting. PLOS ONE. 2012;7: e50568. doi:10.1371/journal.pone.0050568

6. Wessells KR, Singh GM, Brown KH. Estimating the global prevalence of inadequate zinc intake from national food balance sheets: effects of methodological assumptions. PLOS ONE. 2012;7: e50565. doi:10.1371/journal.pone.0050565

7. Beal T, Massiot E, Arsenault JE, Smith MR, Hijmans RJ. Global trends in dietary micronutrient supplies and estimated prevalence of inadequate intakes. PLOS ONE. 2017;12: e0175554. doi:10.1371/journal.pone.0175554

8. Kassebaum NJ, Jasrasaria R, Naghavi M, Wulf SK, Johns N, Lozano R, et al. A systematic analysis of global anemia burden from 1990 to 2010. Blood. 2014;123: 615–624.

9. Institute of Medicine Subcommittee on Interpretation and Uses of Dietary Reference Intakes, Institute of Medicine Standing Committee on the Scientific Evaluation of Dietary Reference Intakes. DRI dietary reference intakes: applications in dietary assessment. Washington (DC): National Academies Press; 2000 [cited 2018 May 16]. Available from: https://www.ncbi.nlm.nih.gov/books/NBK222890/.

10. World Health Organization. Global health observatory (GHO) data [Internet]. 2016 [cited 15 Sep 2016]. Available: http://www.who.int/gho/en/

11. Institute for Health Metrics and Evaluation. GHDx: GBD results tool. Seattle: Institute for Health Metrics and Evaluation; 2013 [cited 2016 Sep 15]. Available from: http://ghdx.healthdata.org/gbd-results-tool.

12. World Health Organization. Global Burden of Disease 2004 update: disability weights for diseases and conditions. Geneva: World Health Organization; 2008 [cited 2018 May 16]. Available from: http://www.who.int/healthinfo/global_burden_disease/GBD2004_DisabilityWeights.pdf?ua=1.

13. Caulfield LE, Black RE. Zinc deficiency. In: Ezzati M, Lopez AD, Rodgers A, Murray CJL, editors. Comparative quantification of health risks: global and regional burden of disease attributable to selected major risk factors. Volume 1. Geneva: World Health Organization; 2004 [cited 2018 May 16]. pp. 257–279. Available from: https://books.google.com/books?hl=en&lr=&id=ACV1jEGx4AgC&oi=fnd&pg=PA257&dq=+Zinc+deficiency+Laura+E.+Caulfield+and+Robert+E.+Black&ots=tXEYvYvZW0&sig=KjRYgW2uv9kc9ROAZTSuuUTxaMo.

14. Myers SS, Zanobetti A, Kloog I, Huybers P, Leakey ADB, Bloom AJ, et al. Increasing CO_2_ threatens human nutrition. Nature. 2014;510:139–42. doi: 10.1038/nature13179

15. Myers SS, Wessells KR, Kloog I, Zanobetti A, Schwartz J. Effect of increased concentrations of atmospheric carbon dioxide on the global threat of zinc deficiency: a modelling study. The Lancet Global Health. 2015;3: e639–e645. doi:10.1016/S2214-109X(15)00093-5

16. Smith MR, Golden CD, Myers SS. Potential rise in iron deficiency due to future anthropogenic carbon dioxide emissions. GeoHealth. 2017;1: 248–257. doi:10.1002/2016GH000018
