# Supplementary material for: Anticipated burden and mitigation of carbon-dioxide-induced nutritional deficiencies and related diseases: A simulation modeling study
Source: PLoS Med. 2018 Jul 3;15(7):e1002586. doi: 10.1371/journal.pmed.1002586 (PMC6029750; doi:10.1371/journal.pmed.1002586)
Supplement: S2 Text — (DOCX) [file pmed.1002586.s021.docx]

**S2 Text: Model structure**

*Model population*

The model evaluated all countries subject to data availability (137 countries). See **S1 Fig** for a map of modeled countries. The model aggregated results from individual countries to compute results globally and for each WHO region, which include the African Region, Region of the Americas, South-East Asia Region, European Region, Eastern Mediterranean Region, and Western Pacific Region. We accounted for demographic- and country-specific changes in population sizes from 2015 to 2050 using secular birth/death trend projections from WHO and the GBD [1,2].

For each country, the model is initialized with a demographically representative set of 10,000 people and run 10,000 times. The model estimated risk factors for each person including age (0-1 year, 1-4 years, and then by 5 year increments), sex, zinc intake, and iron intake. The model included six potential actions for an individual during each annual time step: 1) births, 2) updating risk factors, 3) updating deficiency statuses, 4) updating disease statuses, 5) deaths, and 6) applying mitigation strategies if applicable. Cause-specific YLDs, YLLs, and DALYs were calculated for each person.

*Risk factors*

Risk factors were updated yearly for each person in the model. Zinc intakes decreased by the yearly decrease in average zinc intake for the country, which was calculated with data from the FAOSTAT, USDA, and Myers et al. assuming micronutrient concentrations declined linearly with rising carbon dioxide concentrations [3–5]. Iron intakes decreased by the yearly decrease in average iron intake for the country, which was calculated analogously.

*Transitions*

Health states in the model included healthy, zinc deficient, iron deficient, malaria, pneumonia, diarrheal illness, iron deficiency anemia, and dead. Zinc deficiency only increased the risk of and from malaria, pneumonia, and diarrheal illness in children under 5 years of age as it has not been robustly shown to increase morbidity or mortality from these diseases in adults [6]. During each cycle, a person could only be in one set of health states.

Health states for each person were assigned at the start of the model and after each cycle based on risk factors. Zinc and iron deficiencies were assigned with the estimated average requirement cut-point method [7–9]. This method allowed for estimation of the number of people in a population with intakes less than the requirement. All iron-deficient people were assigned iron deficiency anemia as the iron WtdEARs were calculated from estimates of the prevalence rates of iron deficiency anemia. Each remaining disease status (malaria, pneumonia, and diarrhea) were stochastically estimated with a binomial probability function with probability equal to disease risk as a function of the person’s demographic group and zinc deficiency status. Deaths were similarly determined by a binomial probability function based on mortality rates for a person’s demographic group and deficiency statuses.

*Disease burden*

The model calculated disease burden in net present DALYs, which are the sum of years lived with disability and years of life lost, discounted at an annual rate of 3% (**S4 Table**). Results by decadal period are shown in **S9 Fig**.

**S2 Text References**

1. World Health Organization. Global health observatory (GHO) data [Internet]. 2016 [cited 15 Sep 2016]. Available: http://www.who.int/gho/en/

2. Institute for Health Metrics and Evaluation. GHDx: GBD results tool. Seattle: Institute for Health Metrics and Evaluation; 2013 [cited 2016 Sep 15]. Available from: http://ghdx.healthdata.org/gbd-results-tool.

3. Food and Agriculture Organization of the United Nations. FAOSTAT: food balance sheets. Rome: Food and Agriculture Organization of the United Nations; 2013 [cited 2016 Sep 15]. Available from: http://www.fao.org/faostat/en/#data/FBS.

4. US Department of Agriculture. USDA National Nutrient Database for Standard Reference, Release 28. 2016 [cited 15 Sep 2016]. Available from: https://www.ars.usda.gov/northeast-area/beltsville-md-bhnrc/beltsville-human-nutrition-research-center/nutrient-data-laboratory/docs/usda-national-nutrient-database-for-standard-reference/.

5. Myers SS, Zanobetti A, Kloog I, Huybers P, Leakey ADB, Bloom AJ, et al. Increasing CO_2_ threatens human nutrition. Nature. 2014;510: 139–142. doi:10.1038/nature13179

6. Caulfield LE, Black RE. Zinc deficiency. In: Ezzati M, Lopez AD, Rodgers A, Murray CJL, editors. Comparative quantification of health risks: global and regional burden of disease attributable to selected major risk factors. Volume 1. Geneva: World Health Organization; 2004 [cited 2018 May 16]. pp. 257–279. Available from: https://books.google.com/books?hl=en&lr=&id=ACV1jEGx4AgC&oi=fnd&pg=PA257&dq=+Zinc+deficiency+Laura+E.+Caulfield+and+Robert+E.+Black&ots=tXEYvYvZW0&sig=KjRYgW2uv9kc9ROAZTSuuUTxaMo.

7. Carriquiry AL. Assessing the prevalence of nutrient inadequacy. Public Health Nutr. 1999;2: 23–33.

8. Stadlmayr B, Wijesinha-Bettoni R, Haytowitz D, Rittenschober D, Cunningham J, Sobolewski R, et al. FAO/INFOODS guidelines: guidelines for food matching. Version 1.2. Rome: Food and Agriculture Organization of the United Nations; 2012 [cited 2018 May 16]. Available from: http://www.fao.org/infoods/infoods/standards-guidelines/en/.

9. Wuehler SE, Peerson JM, Brown KH. Use of national food balance data to estimate the adequacy of zinc in national food supplies: methodology and regional estimates. Public Health Nutr. 2005;8: 812–819.
