# Supplementary material for: Anticipated burden and mitigation of carbon-dioxide-induced nutritional deficiencies and related diseases: A simulation modeling study
Source: PLoS Med. 2018 Jul 3;15(7):e1002586. doi: 10.1371/journal.pmed.1002586 (PMC6029750; doi:10.1371/journal.pmed.1002586)
Supplement: S3 Text — (DOCX) [file pmed.1002586.s022.docx]

**S3 Text: Model validation**

Three sets of model results were validated for all populations including intermediate results, one-year disease burden results, and 35-year disease burden results as defined below. We ensured that results showed good concordance with literature values, as described below, at both the country and region levels, before using the model for DALY estimation.

For intermediate results, modeled zinc and iron supplies and percent deficiencies in 2015 were compared to estimates from Kumssa et al. and Kassebaum et al. [1,2] (**S1 Table).** For one-year disease burden results, modeled YLDs, YLLs, and deaths from malaria, pneumonia, diarrhea, and iron deficiency anemia in 2015 were compared to estimates from the GBD [3] (**S2 Table**). For 35-year disease burden results, modeled YLDs, YLLs, and deaths from malaria, pneumonia, diarrhea, and iron deficiency anemia from 2015 to 2050 were compared to estimates from the GBD [3] (**S3 Table**).

The input data for the model and the validation data were partially but not completely overlapping. GBD data was used in both the model and validation [3]. However, in our model, we used their stratified estimates (e.g. per capita, age, and gender); to validate our model, we generally used their aggregate, country-level estimates. Thus, we needed to combine input data from the GBD with other fully independent data sources and use simulation modeling to arrive at aggregate country-level estimates. The GBD country-level aggregate estimates used for validation were derived from other methods, not involving projections from simulation modeling but rather contemporaneous Bayesian statistics. For validation of zinc supplies and deficiency rates, we used the same WtdEARs as Kumssa et al. [2], however, our estimates were otherwise independent. For validation of iron percent deficiencies, we used WtdEARs based on Kassebaum et al. [1].

**S3 Text References**

1. Kassebaum NJ, Jasrasaria R, Naghavi M, Wulf SK, Johns N, Lozano R, et al. A systematic analysis of global anemia burden from 1990 to 2010. Blood. 2014;123: 615–624.

2. Kumssa DB, Joy EJM, Ander EL, Watts MJ, Young SD, Walker S, et al. Dietary calcium and zinc deficiency risks are decreasing but remain prevalent. Sci Rep. 2015;5:10974.

3. Institute for Health Metrics and Evaluation. GHDx: GBD results tool. Seattle: Institute for Health Metrics and Evaluation; 2013 [cited 2016 Sep 15]. Available from: http://ghdx.healthdata.org/gbd-results-tool.
