# Supplementary material for: Anticipated burden and mitigation of carbon-dioxide-induced nutritional deficiencies and related diseases: A simulation modeling study
Source: PLoS Med. 2018 Jul 3;15(7):e1002586. doi: 10.1371/journal.pmed.1002586 (PMC6029750; doi:10.1371/journal.pmed.1002586)
Supplement: S4 Text — (DOCX) [file pmed.1002586.s023.docx]

**S4 Text: Sensitivity analysis**

Probabilistic sensitivity analysis was performed for all countries to test the robustness of projected disease burden to changes in combinations of inputs. Distributions were constructed for model inputs (**S5 Table**) and the model was run 10,000 times for each country while sampling from each probability distribution of each parameter. Mean net present DALYs from 2015 to 2050 and 95% credible intervals are shown in **S6 Table**.

One-way sensitivity analyses were also performed across all model inputs (**S2-S8 Figs**).

We evaluated how using lognormal iron consumption distributions with 40% CVs instead of normal iron consumption distributions with 25% CVs would affect model results. The model was run 10,000 times for each country while sampling from distributions reflecting uncertainty in model parameters as previously described. Results from this sensitivity analysis are shown in **S7 Table** and can be compared to the baseline model results in **S6 Table**; changes in iron consumption distributions did not materially affect model results.

Finally, we evaluated how constant disease prevalence rates would affect model results. Malaria, pneumonia, and diarrhea prevalence rates in 2015 were assumed to continue over the model period. The model was run 10,000 times for each country while sampling from distributions reflecting uncertainty in model parameters as previously described. Results from this sensitivity analysis are shown in **S8** **Table** and can be compared to the baseline model results in **S6 Table**; constant disease prevalence rates did not materially affect model results.
