# Supplementary material for: Anticipated burden and mitigation of carbon-dioxide-induced nutritional deficiencies and related diseases: A simulation modeling study
Source: PLoS Med. 2018 Jul 3;15(7):e1002586. doi: 10.1371/journal.pmed.1002586 (PMC6029750; doi:10.1371/journal.pmed.1002586)
Supplement: S5 Text — (DOCX) [file pmed.1002586.s024.docx]

**S5 Text: Mitigation strategies**

Six mitigation strategies were evaluated for each country, including climate mitigation, zinc supplementation, iron supplementation, malaria mitigation, pneumonia mitigation, and diarrheal illness mitigation. The climate mitigation strategy involved countries adhering to the recent Paris Agreement and keeping temperatures within 2°C of pre-industrial levels. The Paris Agreement is expected to prevent approximately 47% of the increase in carbon dioxide concentration by 2050 [1,2]. Since the 2015 carbon dioxide concentration was approximately 400 ppm and the baseline 2050 carbon dioxide concentration is estimated to be approximately 550 ppm [3–6], following the Paris Agreement is expected to lead to a 2050 carbon dioxide concentration of approximately 480 ppm. The zinc supplementation strategy involved daily zinc supplementation of 80% of children under 5 years of age (randomly selected) with 10 mg/day of zinc [7]. The iron supplementation strategy involved weekly iron supplementation of 80% of females over 5 years of age (randomly selected) with 60 mg/week of elemental iron in the form of ferrous sulphate [8]. The malaria mitigation strategy involved adoption of a previously described portfolio of interventions at an 80% coverage level including long-lasting insecticide treated nets (LLINs), indoor residual spraying (IRS), and mass screening and treatment (MSAT) [9]. Specifically, settings with low entomological inoculation rates (EIRs) just received LLINs; settings with moderate EIRs received LLINs, IRS, and MSAT; and settings with high EIRs received LLINs, IRS, and MSAT in addition to social improvements. The pneumonia mitigation strategy involved adoption of a previously described portfolio of interventions at an 80% coverage level (except for vaccines, which were adopted at a 90% coverage level) including *Haemophilus influenzae* type b vaccine, pneumococcal vaccine, antibiotics for pneumonia, promotion of breastfeeding, vitamin A supplementation, improved water source, water connection in the home, improved excreta disposal (latrine or toilet), and hygienic disposal of children’s stools [10]. The diarrheal illness mitigation strategy involved adoption of a previously described portfolio of interventions at an 80% coverage level (except for the vaccine, which was adopted at a 90% coverage level) including oral rehydration solution, rotavirus vaccine, antibiotics for dysentery, promotion of breastfeeding, vitamin A supplementation, improved water source, water connection in the home, improved excreta disposal (latrine or toilet), and hygienic disposal of children’s stools [10]. See results for regions in **S10 Fig** and results for each country in **S9 Table**.

**S5 Text References**

1. Meinshausen M, Smith SJ, Calvin K, Daniel JS, Kainuma MLT, Lamarque J-F, et al. The RCP greenhouse gas concentrations and their extensions from 1765 to 2300. Climatic Change. 2011;109: 213. doi:10.1007/s10584-011-0156-z

2. Rogelj J, Meinshausen M, Knutti R. Global warming under old and new scenarios using IPCC climate sensitivity range estimates. Nature Clim Change. 2012;2: 248–253. doi:10.1038/nclimate1385

3. Earth System Research Laboratory Global Monitoring Division. Global Greenhouse Gas Reference Network. Trends in atmospheric carbon dioxide. Data: Mauna Loa CO_2_ annual mean data. Boulder: Earth System Research Laboratory; 2018 [cited 2018 Mar 24]. Available from: https://www.esrl.noaa.gov/gmd/ccgg/trends/data.html.

4. Myers SS, Zanobetti A, Kloog I, Huybers P, Leakey ADB, Bloom AJ, et al. Increasing CO_2_ threatens human nutrition. Nature. 2014;510: 139–142. doi:10.1038/nature13179

5. Myers SS, Wessells KR, Kloog I, Zanobetti A, Schwartz J. Effect of increased concentrations of atmospheric carbon dioxide on the global threat of zinc deficiency: a modelling study. The Lancet Global Health. 2015;3: e639–e645. doi:10.1016/S2214-109X(15)00093-5

6. Fisher BS, Nakicenovic N, Alfsen K, Morlot JC, Chesnaye F, Hourcade J-C, et al. Issues related to mitigation in the long term context. In: Metz B, Davidson OR, Bosch PR, Dave R, Meyer LA, editors. Climate change 2007: mitigation of climate change. Working Group III contribution to the fourth assessment report of the Intergovernmental Panel on Climate Change. Cambridge: Cambridge University Press; 2007.

7. Yakoob MY, Theodoratou E, Jabeen A, Imdad A, Eisele TP, Ferguson J, et al. Preventive zinc supplementation in developing countries: impact on mortality and morbidity due to diarrhea, pneumonia and malaria. BMC Public Health. 2011;11(Suppl 3):S23. doi: 10.1186/1471-2458-11-S3-S23

8. Pasricha S-R, Drakesmith H, Black J, Hipgrave D, Biggs B-A. Control of iron deficiency anemia in low- and middle-income countries. Blood. 2013;121: 2607–2617. doi:10.1182/blood-2012-09-453522

9. Griffin JT, Hollingsworth TD, Okell LC, Churcher TS, White M, Hinsley W, et al. Reducing plasmodium falciparum malaria transmission in Africa: a model-based evaluation of intervention strategies. PLOS Med. 2010;7: e1000324. doi:10.1371/journal.pmed.1000324

10. Bhutta ZA, Das JK, Walker N, Rizvi A, Campbell H, Rudan I, et al. Interventions to address deaths from childhood pneumonia and diarrhoea equitably: what works and at what cost? Lancet. 2013;381: 1417–1429. doi:10.1016/S0140-6736(13)60648-0
